# Tracing NAD^+^ metabolism uncovers adaptive coordination between host and microbiome during colitis

**DOI:** 10.1016/j.celrep.2026.117730

**Published:** 2026-07-25

**Authors:** Abrar I. Alsaadi, Lina Wehkamp, Anant A. Pothakamury, Mahmoud S. Yahia, Praveena Prasad, Clara Gilloteau, Jaedon Sadler, Jaclyn D. Smith, Brenita C. Jenkins, Garret S. Diven, Johanna Bornhäuser, Taous Mekdoud, Shuchang Tian, Philip Rosenstiel, Stefan Schreiber, Jordan E. Bisanz, Konrad Aden, Melanie R. McReynolds

**Affiliations:** 1 Department of Biochemistry and Molecular Biology, The Huck Institute of Life Sciences, Pennsylvania State University, University Park, PA, USA; 2 Institute of Clinical Molecular Biology, Christian-Albrechts-University and University Hospital Schleswig-Holstein, Campus Kiel, Kiel, Germany; 3 Department of Internal Medicine L., Christian-Albrechts-University and University Hospital Schleswig-Holstein, Campus Kiel, Kiel, Germany; 4 These authors contributed equally; 5 Lead contact

## Abstract

Host-microbiota metabolic interactions critically regulate nicotinamide adenine dinucleotide (NAD^+^) homeostasis, and their disruption is increasingly linked to chronic diseases, including inflammatory bowel disease (IBD). However, it remains unclear whether NAD^+^ dysregulation in IBD arises from impaired production, enhanced consumption, or both. Using multi-omics approaches and stable isotope-labeled NAD^+^ precursors administered via intravenous infusion in a murine model of dextran sulfate sodium (DSS)-induced colitis, we mapped tissue- and lumen-specific NAD^+^ metabolism under inflammatory stress. Our results reveal tissue-specific rewiring of NAD^+^ metabolism, with increased flux through the salvage pathway compensating for reduced *de novo* NAD^+^ synthesis from tryptophan. In parallel, microbial *de novo* NAD^+^ production was elevated, highlighting a cooperative host-microbiota response to inflammatory stress. These findings demonstrate differential regulation of NAD^+^ biosynthesis during acute colitis and underscore the dynamic interplay between host and microbial metabolism in maintaining NAD^+^ homeostasis under inflammatory conditions.

## In brief

Using isotope tracing and metabolomics, Alsaadi and colleagues show that colitis disrupts NAD^+^ metabolism in a pathway-specific manner, triggering compensatory salvage pathway activation in host tissues and distinct metabolic adaptations across the microbiome. These findings reveal coordinated host-microbe metabolic remodeling during inflammatory stress and highlight NAD^+^ metabolism as a dynamic mediator of intestinal disease.

## Graphical Abstract

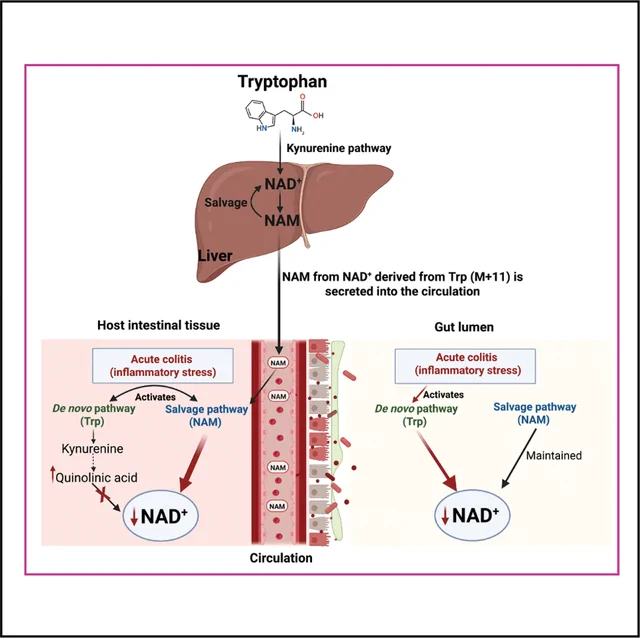

## INTRODUCTION

Interactions between the host and the gut microbiota are critical for maintaining nicotinamide adenine dinucleotide (NAD^+^) homeostasis.^1^ NAD^+^ is a central coenzyme that regulates cellular metabolism and signaling, supporting processes such as energy production, DNA repair, and stress responses.^2–4^ In mammals, NAD^+^ can be synthesized *de novo* from tryptophan (Trp) through the kynurenine pathway (KP), Preiss-Handler pathway from nicotinic acid (NA), from nicotinamide riboside (NR), but most of the NAD^+^ synthesis is derived from nicotinamide (NAM) through the salvage pathway.^5,6^ Despite its importance, the regulation and utilization of these pathways under pathological conditions remain poorly understood.

Recent studies highlight the gut microbiota’s role in supporting host NAD^+^ homeostasis through the bidirectional cycling of NAD^+^ precursors between the host tissues and the gut microbiota under normal physiological conditions.^1,7^ The gut microbiota contributes to host NAD^+^ synthesis by converting host-derived NAM to NA via bacterial nicotinamidase encoded by the *PncA* gene, a process not found in mammals. Thus, the presence of NA in the gastrointestinal (GI) tract is microbiome-dependent, which provides an alternative route for NAD^+^ synthesis in the host tissues and gut microbiota.^1,7^ However, physiological stressors, such as infection or inflammation, can disrupt this bidirectional metabolic communication, leading to dysregulation of NAD^+^ biosynthesis and degradation pathways and potentially contributing to disease pathology.

Emerging evidence has linked altered NAD^+^ metabolism to intestinal inflammation, suggesting its dysregulation may contribute to the pathogenesis of inflammatory bowel disease (IBD) by altering immune function and tissue repair.^8–12^ IBD, including Crohn’s disease (CD) and ulcerative colitis (UC), is a chronic inflammatory condition of the GI tract, leading to tissue damage, gut dysbiosis, and an altered gut environment.^13^ While significant progress has been made in understanding the immune and genetic mechanisms of IBD, the metabolic reprogramming that accompanies disease manifestation and progression remains poorly understood, despite its potential to drive disease pathology.^14–16^ Therefore, we hypothesized that acute colitis disrupts NAD^+^ homeostasis in both the host tissues and the gut microbiota, resulting in increased NAD^+^ turnover driven by heightened inflammation-induced energy demand.

While the role of NAD^+^ in inflammation is recognized, there is limited understanding of how acute inflammation, such as that seen in dextran sulfate sodium (DSS)-induced colitis, alters NAD^+^ biosynthesis and its pathways in a tissue-specific manner. In this study, we leverage a multi-omics approach using isotope tracing via intravenous infusions, metabolomics, and metagenomics to explore how the host and the gut microbiome coordinate NAD^+^ biosynthesis under normal physiological and inflammatory conditions. Using stable isotope tracers of Trp and nicotinamide, we aimed to determine how acute colitis reprograms NAD^+^ metabolism across different tissues and within the gut microbiome, with particular emphasis on the activation of *de novo* and salvage biosynthetic pathways.

By integrating complementary omics approaches, we identified dynamic metabolic changes that disrupt NAD^+^ homeostasis and contribute to disease progression. Our analysis demonstrates that acute DSS-induced colitis rewires NAD^+^ metabolism, activating nicotinamide (NAM)-driven salvage pathways in a tissue-specific manner as a compensatory response to sustain NAD^+^ levels during intestinal inflammation. This previously unrecognized connection between dysregulated NAD^+^ metabolism and intestinal inflammation highlights potential therapeutic avenues to restore metabolic balance, improve intestinal health, and enhance patient outcomes.

## RESULTS

### Inflammation and tissue damage associate with disrupted host and microbial NAD^+^ metabolism in acute colitis

**Figure 1.**
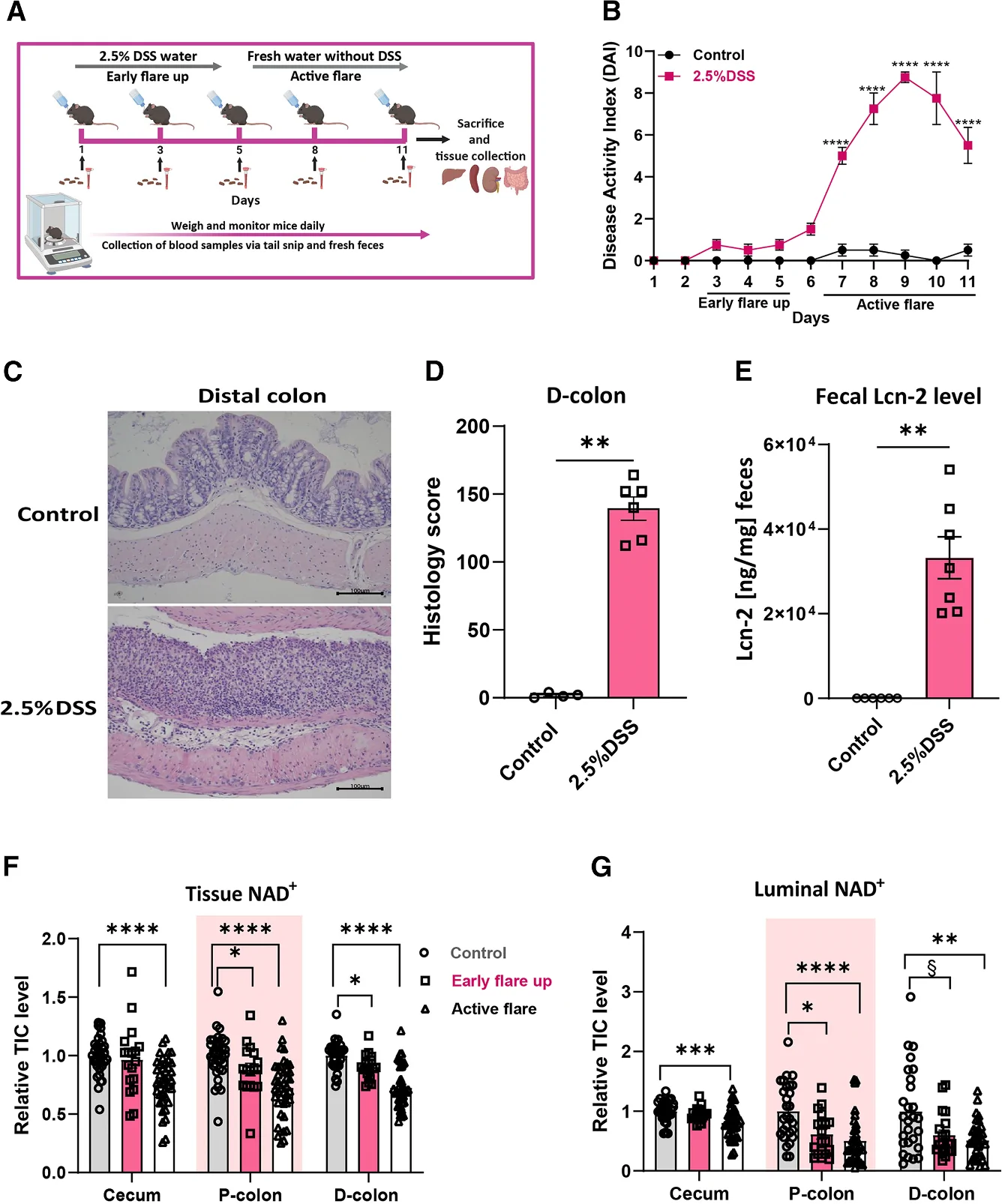
Acute-induced colitis decreases NAD^+^ levels in colonic tissues (A) Experimental setup illustrating the administration of 2.5% DSS dissolved in water for 5 days, followed by a switch to normal drinking water until the end of the experiment (day 11) in 11- to 12-week-old C57BL/6 male mice. Longitudinal sampling of fecal and blood samples (shown in black arrows) was collected for LC-MS metabolomics and metagenomics analysis. At the end of the experiment, mice were sacrificed by cervical dislocation, tissues and luminal samples were dissected for LC-MS metabolomics. (B) Daily recorded disease activity index (DAI) in DSS-treated and control mice including weight loss compared to the initial weight, stool consistency, bloody stool, rectal bleeding, overall activity, posture, and fur. (C and D) Representative image (C) of the H&E staining of Swiss roll sections of the distal colon segment (at magnification 20×, scale bars, 100 μm) and (D) a corresponding histopathological score. (E) Fecal lcn-2 was measured by ELISA in fecal samples collected on days 8 and 11 during the flare phase of intestinal inflammation and controls. (F and G) LC-MS relative total ion count (TIC) levels of NAD^+^ pooled from all experiments in this study of (F) tissues NAD^+^ levels and (G) luminal NAD^+^ levels. Data are presented as mean ± SEM; in (F), *n* = 18–40; in (G), *n* = 20–40. Statistical significance was determined by Kruskal-Wallis test with post hoc Dunn’s test for comparisons among more than two groups and Mann-Whitney U test for two-group comparisons. § <0.1, *p < 0.05, **p < 0.01, ***p < 0.001, and ****p < 0.0001. (A) was created with Biorender.com.

Acute colitis was induced in mice by administering 2.5% DSS in drinking water for 5 days, followed by normal water until day 11 (Figure 1A).^17,18^ This approach allowed us to monitor disease activity and identify how flux of NAD^+^ is impacted during the flare-up (early) and flare (active) phases of acute induced colitis. The early flare-up phase is characterized by mild colitis occurring on days 4–5, and the active flare phase spans from days 7–11, in which the colon is fully inflamed^19–21^ (Figure S1A). DSS-treated mice showed a substantial weight loss (Figure S1B), and increased disease activity index (DAI) during the active flare phase compared to the early flare-up phase and control mice^18,22^ (Figure 1B). The induction of acute colitis resulted in structural alterations of the epithelial and mucosal layer in the distal colon (D-colon) of DSS-treated mice, leading to a higher score for histological inflammation compared to control mice (Figures 1C and 1D). To further confirm the induction of colitis, we measured the levels of fecal lipocalin-2 (Lcn-2), also known as neutrophil gelatinase-associated lipocalin (NGAL), a biomarker of intestinal inflammation.^23,24^ Fecal Lcn-2 levels were significantly elevated in mice treated with 2.5% DSS compared to control mice, reflecting increased intestinal inflammation, which was further supported by the upregulation of Lcn-2 expression in the D-colon (Figures 1E and S1C). We also observed a significant upregulation of pro-inflammatory cytokines, including *Ifn-γ*, *Tnf-α*, *Il-6*, and *Il-1β*, while *Il-1α* showed an upward trend (Figures S1D–S1H). We noted elevated levels of anti-inflammatory cytokines *Il-10* and *Il-22* in DSS-treated mice compared to controls in the D-colon (Figures S1I and S1J). Supporting previous observations,^25^ the total NAD^+^ pool was reduced in the cecum and colon during intestinal inflammation (Figure 1F). Additionally, DSS treatment in the active flare phase resulted in disrupted luminal NAD^+^ levels in the cecum and colon, indicating disrupted microbial NAD^+^ metabolism (Figure 1G).

### Increased NAD^+^ synthesis from Trp in acute induced colitis

Trp is an essential amino acid metabolized via the KP, leading to the production of quinolinic acid (QUIN) and subsequent NAD^+^ synthesis (Figure S2A). The KP is primarily mediated by hepatic tryptophan 2,3-dioxygenase (TDO), with a lesser contribution from extrahepatic indoleamine 2,3-dioxygenase (IDO).^26,27^ Previous studies have indicated that IBD is associated with alterations in Trp metabolism, which result in increased conversion of Trp to Kyn due to elevated IDO enzyme activity and is reflected by an increased Kyn/Trp ratio.^11,12,28,29^ Supporting this, we detected an increase of the circulating Kyn/Trp ratio during both the early flare-up and active flare phases compared to control^30^ (Figure S2B). We hypothesized that the increased Trp flux through the KP represents a compensatory response to inflammation, in which NAD^+^ demand is heightened during the immune response.

**Figure 2.**
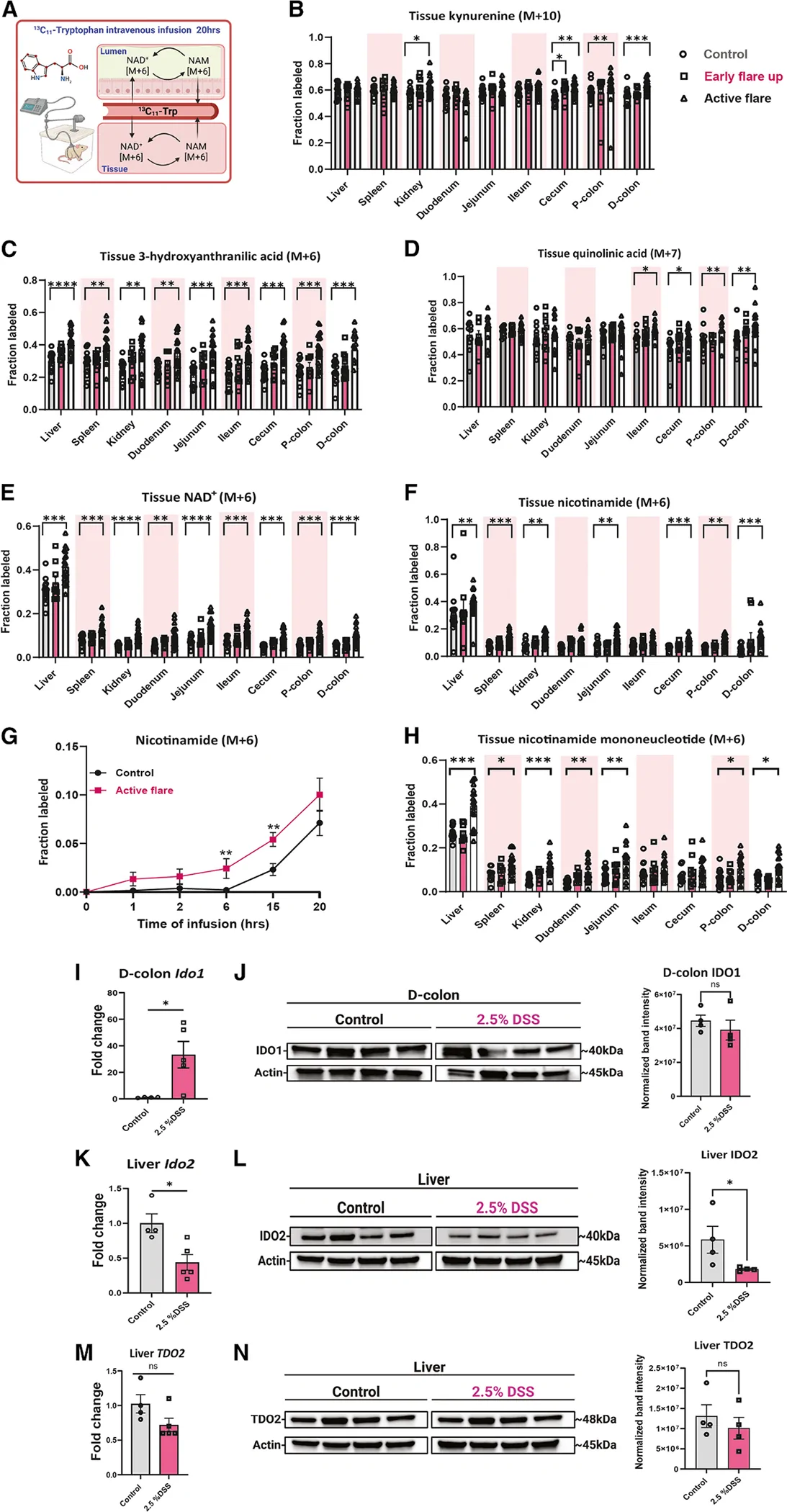
Enhanced NAD^+^ production from tryptophan during acute DSS-induced intestinal inflammation (A) Experimental schematic of intravenous infusion of universally labeled tryptophan (^13^C_11_-Trp) into pre-catheterized male C57BL/6 mice (11–12 weeks old) to assess NAD^+^ flux from tryptophan in host tissues and luminal samples. (B–F) Tissue fractional labeling following 20-h intravenous infusion of (B) kynurenine, (C) 3-hydroxyanthranilic acid, (D) quinolinic acid, (E) NAD^+^, and (F) nicotinamide. (G) Serum fractional labeling of nicotinamide over 20-h intravenous infusion during the active flare phase of intestinal inflammation. (H) Tissue fractional labeling following 20-h intravenous infusion of nicotinamide mononucleotide. (I) Distal colon; indoleamine 2,3-dioxygenase 1 (*Ido1*) mRNA expression was measured by RT-qPCR and normalized to TATA-box binding protein (*Tbp*) during the active flare phase (days 8 and 11) compared to control mice. (J) Representative western blot and quantification of IDO1 protein levels in the distal colon from control and 2.5% DSS-treated mice during the active flare phase (days 8 and 11) normalized to β-actin. (K) Liver; indoleamine 2,3-dioxygenase 1(*Ido2*) mRNA expression was measured by RT-qPCR and normalized to TATA-box binding protein (*Tbp*) during the active flare phase (days 8 and 11) compared to control mice. (L) Representative western blot and quantification of IDO2 protein levels in the liver from control and 2.5% DSS-treated mice during the active flare phase (days 8 and 11) normalized to β-actin. (M) Liver; tryptophan 2,3-dioxygenase (*Tdo2*) mRNA expression was measured by RT-qPCR and normalized to TATA-box binding protein (*Tbp*) during the active flare phase (days 8 and 11) compared to control mice. (N) Representative western blot and quantification of TDO2 protein levels in the liver from control and 2.5% DSS-treated mice during the active flare phase (days 8 and 11) normalized to β-actin. Data are presented as mean ± SEM; *n* = 10–20 in (B)–(H), and *n* = 4–5 in (I)–(N). Statistical significance was determined by Kruskal-Wallis test with post hoc Dunn’s test for comparisons among more than two groups and Mann-Whitney U test for two-group comparisons. NS, not significant, § < 0.1, *p < 0.05, **p < 0.01, ***p < 0.001, and ****p < 0.0001. (A) was created with BioRender.com

To assess whether the flux of Trp to NAD^+^ is altered by colitis induction, we performed *in vivo* isotope tracing using stable isotope-labeled Trp (U-^13^C_11_). The tracer was infused at a constant rate of 1.25 nmol/g body weight/min for 20 h in both DSS-treated and control mice during both the early flare-up and the active flare phases of inflammation (Figure 2A). In the serum, the fraction of labeled Trp (M+11), Kyn (M+10), and Quin (M+7) was not significantly altered between DSS-treated and control mice at different phases of inflammation (Figures S2C–S2E). Moreover, the whole-body analysis of tissues revealed no difference in Trp (M+11) labeling between diseased and control mice (Figure S2F). However, there was an increase in the fraction labeled Kyn (M+10) in the cecum during the early flare-up and in the kidney, cecum, and colon during the active flare phase, suggesting an enhanced flux of Trp via the KP in acute DSS-induced colitis (Figure 2B). This was further confirmed by increased labeling of the downstream metabolites with the expected label, 3-hydroxyanthranilic acid (3HAA, M+6) and QUIN (M+7)^5^ (Figures 2C and 2D). Ultimately, KP-mediated Trp catabolism resulted in elevated NAD^+^ (M+6) production in multiple tissues during the active flare phase, with the highest levels detected in the liver (Figure 2E). This elevation is consistent with the liver’s role as the primary site of Trp metabolism via the *de novo* pathway, where it synthesizes NAD^+^ from Trp and releases NAM (M+6) to support NAD^+^ synthesis in extrahepatic tissues.^5^ The release of NAM (M+6) from NAD^+^ (M+6), which can either be recycled to synthesize NAD^+^ or secreted into the circulation, was elevated in most tissues, including the liver, spleen, kidney, small intestine, and colon, during the active flare phase (Figure 2F). Circulating NAM (M+6) levels increased at 6 h in the active flare but not the early flare-up phase, compared to control (Figures 2G and S2G). This was accompanied by increased levels of labeled nicotinamide mononucleotide (NMN; M+6) across multiple tissues, indicating enhanced NAD^+^ turnover during intestinal inflammation (Figure 2H).

The observed increase in Trp downstream metabolites supports the activation of the KP during intestinal inflammation, which has been previously described to be mediated by inflammatory cytokines and immune cell activation.^31^ Accordingly, colonic gene expression of *Ido1* was upregulated (Figure 2I), while Ido1 protein levels did not show a corresponding significant change (Figure 2J). Interestingly, DSS treatment led to a significant downregulation of *Ido2* transcript and protein levels in the liver (Figures 2I–2L). The liver *Tdo2* transcript and protein levels were not significantly altered in the DSS-treated mice (Figures 2M and 2N). We observed the previously described metabolic blockade at the level of *Qprt* (Figures S2H and S2I). At first glance, the detection of increased Trp-derived NAD^+^ (M+6) flux appears paradoxical. However, because NAD^+^ (M+6) can also originate from nicotinamide (NAM, M+6) recycled through hepatic metabolism, our data suggest that the majority of NAD^+^ (M+6) detected in the colon arises from NAM salvage rather than *de novo* synthesis, which remains impaired due to the *Qprt* blockade.

Overall, these findings reveal tissue-specific rewiring of Trp catabolism in response to colitis, in which Trp fulfills a dual role: fueling *de novo* NAD^+^ synthesis primarily in the liver and supporting hepatic recycling of NAD^+^ to NAM to sustain systemic NAD^+^ homeostasis through the salvage pathway.

### Acute colitis enhances microbial *de novo* NAD^+^ synthesis from Trp

**Figure 3.**
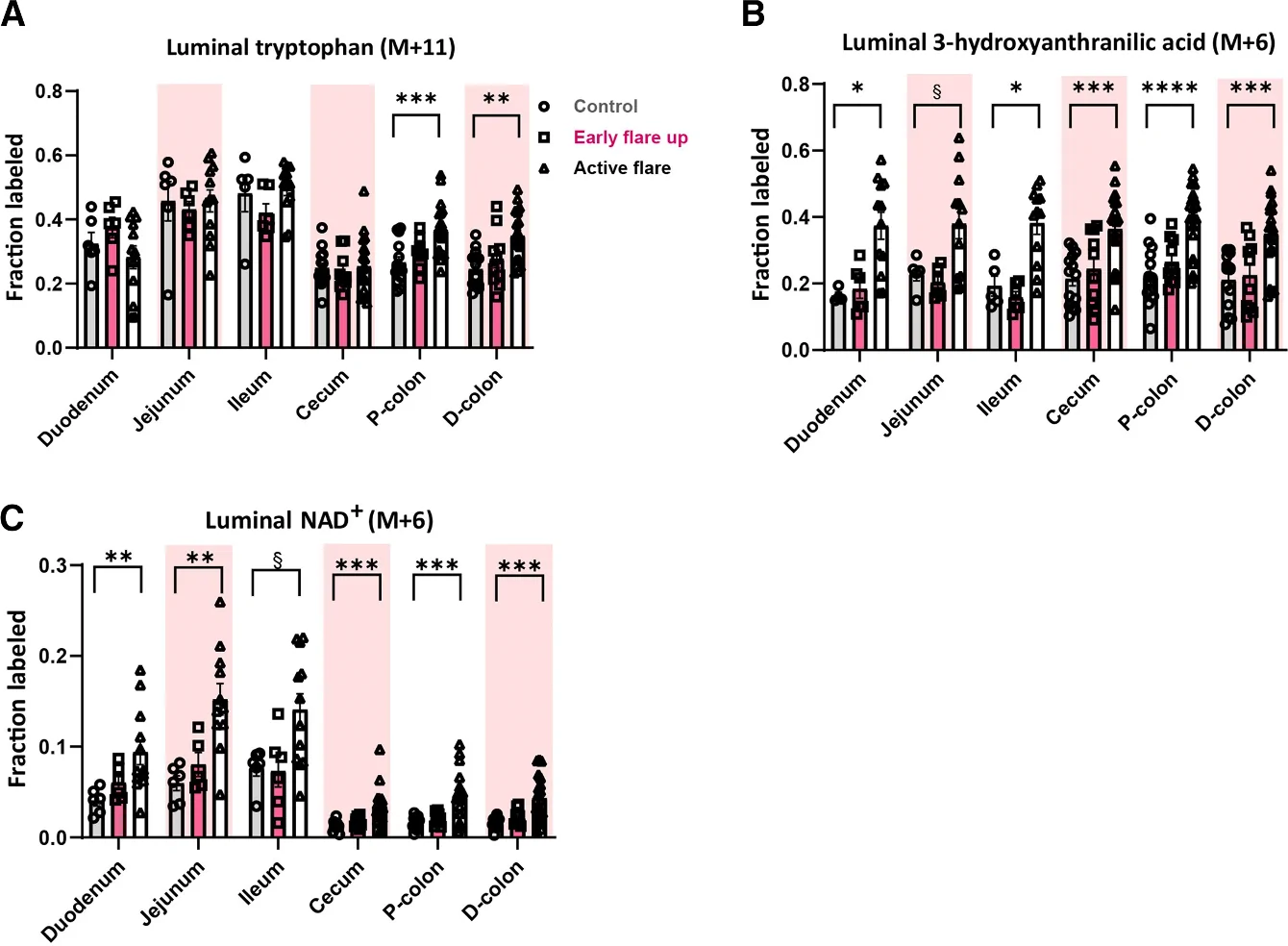
Elevated flux of tryptophan to luminal NAD^+^ during the active flare phase of acute DSS-induced colitis LC-MS luminal fraction of metabolites labeled from the tryptophan tracer (shown in Figure 2) following 20-h intravenous infusion of (A) tryptophan, (B) 3-hydroxyanthranilic acid, and (C) NAD^+^. Data are presented as mean ± SEM (*n* = 10–20). Statistical significance was determined by Kruskal-Wallis test with post hoc Dunn’s test for comparisons among more than two groups. NS, not significant, § < 0.1, *p < 0.05, **p < 0.01, ***p < 0.001, and ****p < 0.0001.

Previous reports highlighted the pivotal role of intestinal microbes in Trp metabolism. Many bacterial species possess enzymes that convert Trp into metabolites essential not only for bacterial functions but also for facilitating key communication pathways between the immune system and the GI tract.^32–34^ Having established that flux of Trp to host NAD^+^ was impacted by intestinal inflammation, we next investigated whether intestinal inflammation similarly disrupts microbial NAD^+^ production in the gut lumen. We measured NAD^+^ levels in the luminal samples collected from different regions of the small intestine and colon, using the tracing approach described in Figure 2. We observed an increase of approximately 15% in Trp (M+11) labeling in the proximal and distal colon lumen during the active flare phase compared to control (Figure 3A). We noted a significant decrease of Kyn (M+10) in the D-colon during the active flare phase (Figure S3A). The fractional labeling of 3HAA (M+6) significantly increased in multiple luminal regions during the active flare phase compared to controls (Figure 3B), while the labeled fraction of QUIN (M+7) remained comparable between DSS-treated and control groups (Figure S3B). Flux of Trp to luminal NAD^+^ (M+6) increased in different luminal regions during the active flare phase compared to control (Figure 3C), with enhanced recycling of NAM (M+6) observed in the duodenum lumen during the active flare phase and in the ileum lumen during the early flare-up phase (Figure S3C). These observations indicate a potentially significant contribution of the gut microbiome to Trp catabolism in acute colitis.

### Colitis-induced alterations of Trp-metabolizing bacteria at the site of inflammation

**Figure 4.**
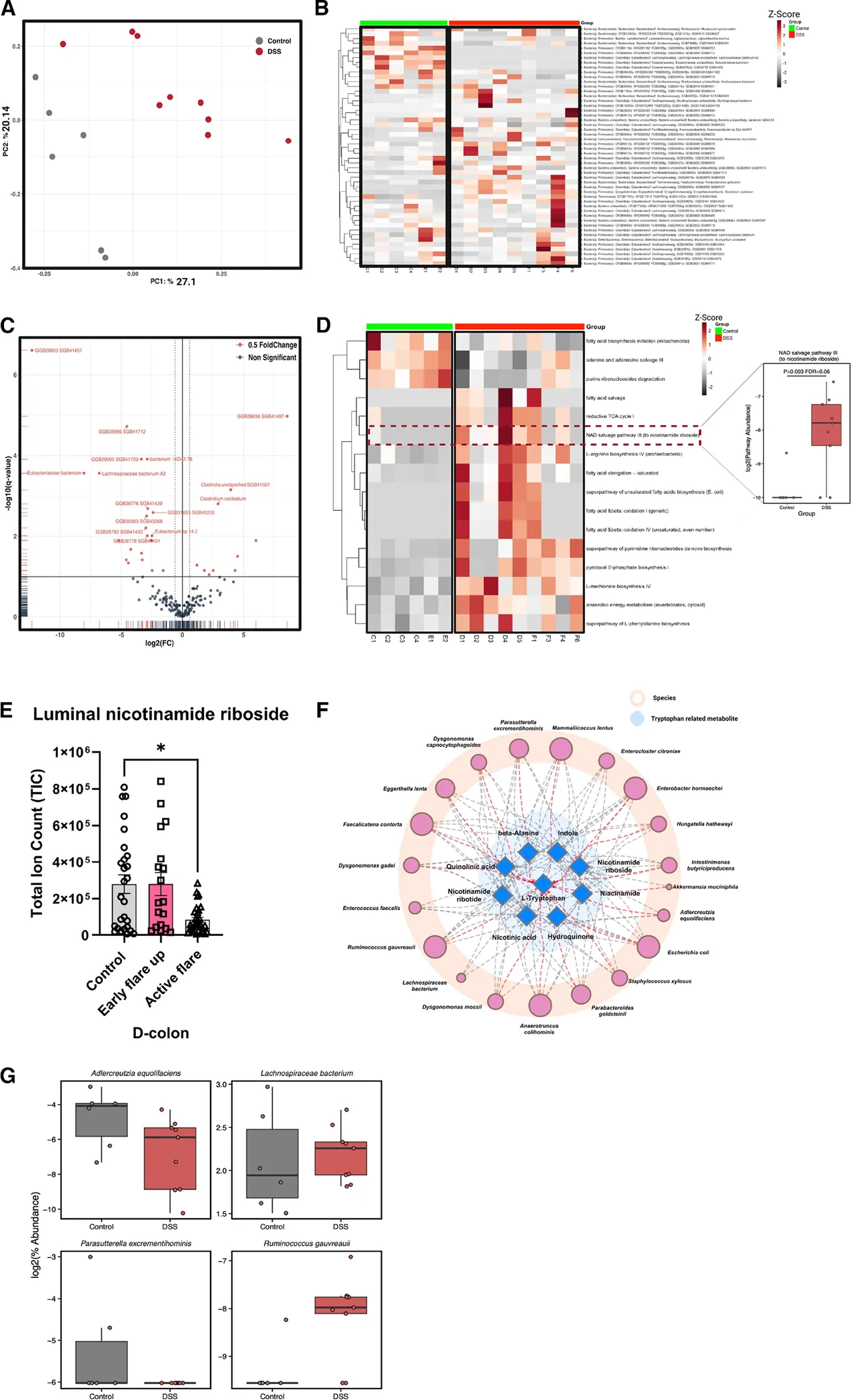
Integrative pathway and taxa analysis of the gut microbiota alterations in acute colitis (A) Bray-Curtis PCoA of species-level profiles shows clear separation between control (gray) and DSS (red) groups during the active phase of colitis (days 8 and 11) (PERMANOVA, R^2^ = 0.216, *p* = 0.002), indicating significant remodeling of the microbial functional potential during DSS-induced colitis. (B) Species-level heatmap of the top 50 most variable taxa, clustered by relative abundance *Z*-scores across samples. Hierarchical clustering reveals distinct community compositions between groups, with several *Clostridium* and *Lachnospiraceae* species enriched in the DSS-treated mice during the active flare phase. (C) Volcano plot of differential species abundance between DSS and control groups. 33 species were significantly altered (FDR < 0.1, |log_2_FC| > 0.5), including 11 species enriched in DSS-treated group and 22 reduced relatives to control. (D) Heatmap of significantly altered metabolic pathways (FDR < 0.1). DSS-induced colitis group shows enrichment of lipid metabolism and the NAD^+^ salvage pathway and depletion of amino acid and purine biosynthetic pathways, suggesting a shift toward energy metabolism and stress-adaptive functions. (E) LC-MS relative total ion count (TIC) levels of luminal nicotinamide riboside in the distal colon pooled from all experiments in this study. (F) Predicted metabolite-taxon network linking 20 microbial taxa to 9 tryptophan-related metabolites via 110 high-confidence edges. (G) Altered tryptophan-metabolizing taxa including four differentially abundant species (*Lachnospiraceae* spp., *Adlercreutzia equolifaciens*, *Ruminococcus gauvreauii*, and *Parabacteroides goldsteinii*), potentially modulating tryptophan metabolism during acute intestinal inflammation.

Having observed increased labeled Trp (M+11) in the colon lumen during the active flare phase, we next investigated whether DSS-induced colitis alters the abundance of Trp-metabolizing bacteria. Metagenomics profiling of the fecal contents revealed a distinct alteration in the gut microbiome composition (Figure 4A) and diversity (Figure S4A) between DSS-treated mice and controls during the active flare phase.^35–37^ Beta diversity analysis of species-level community composition revealed a significant shift with clear stratification by treatment group (Figure 4A, PERMANOVA: R^2^ = 0.2168, *p* = 0.002). DSS treatment was associated with a reduction in observed species but not their distribution (Figure S4B). Differential abundance analysis between DSS-treated and vehicle controls identified 33 species (22 increased in control and 10 increased in DSS-treated; false discovery rate [FDR] ≤ 0.1; Figures 4B and 4C). To understand the metabolic impacts of these taxonomic shifts, we performed pathway analysis, uncovering similar clustering patterns and uncovering 103 differentially abundant pathways (FDR ≤ 0.1, Figures S4C and S4D). Differential pathways representing fatty acid biosynthesis and nucleotide metabolism were enriched in controls (Figure 4D). In line with the observed NAD^+^ metabolic rewiring (Figure 3), metagenomic data uncovered that DSS treatment increased microbial NAD^+^ salvage pathway III, generating NR, suggesting microbial contributions to altered NAD^+^ precursor availability (Figure 4D). However, NR in host tissues was not altered (Figure S4D), whereas luminal NR levels were reduced in the distal colon during the active flare phase compared to control, suggesting that NR abundance reflects the abundance between production and consumption rather than pathway abundance alone (Figure 4E). Owing to the poorly characterized nature of the mouse microbiome, to better understand the organisms associated with altered NAD^+^ metabolism, we integrated species abundances with Trp metabolites from metabolomics, resulting in a predicted network of 389 metabolites, 20 taxa, and 4,070 edges. Zero-order filtering refined this to 9 metabolites and 20 taxa with 110 edges, highlighting species interacting with Trp and its related metabolites via well-annotated pathways (Figure 4F). The network analysis identified 20 bacterial species involved in Trp metabolism during DSS-induced colitis. Of those, 4 species, *Lachnospiraceae* spp., *Adlercreutzia equolifaciens*, *Ruminococcus gauvreauii*, and *Parabacteroides goldsteinii*, were significantly altered between DSS and control mice, suggesting a potential role in modulating Trp metabolism during the active flare phase of acute colitis (Figure 4G).

### Induction of acute colitis drives tissue-specific alterations of Trp-dependent metabolites in the host and the gut microbiota

**Figure 5.**
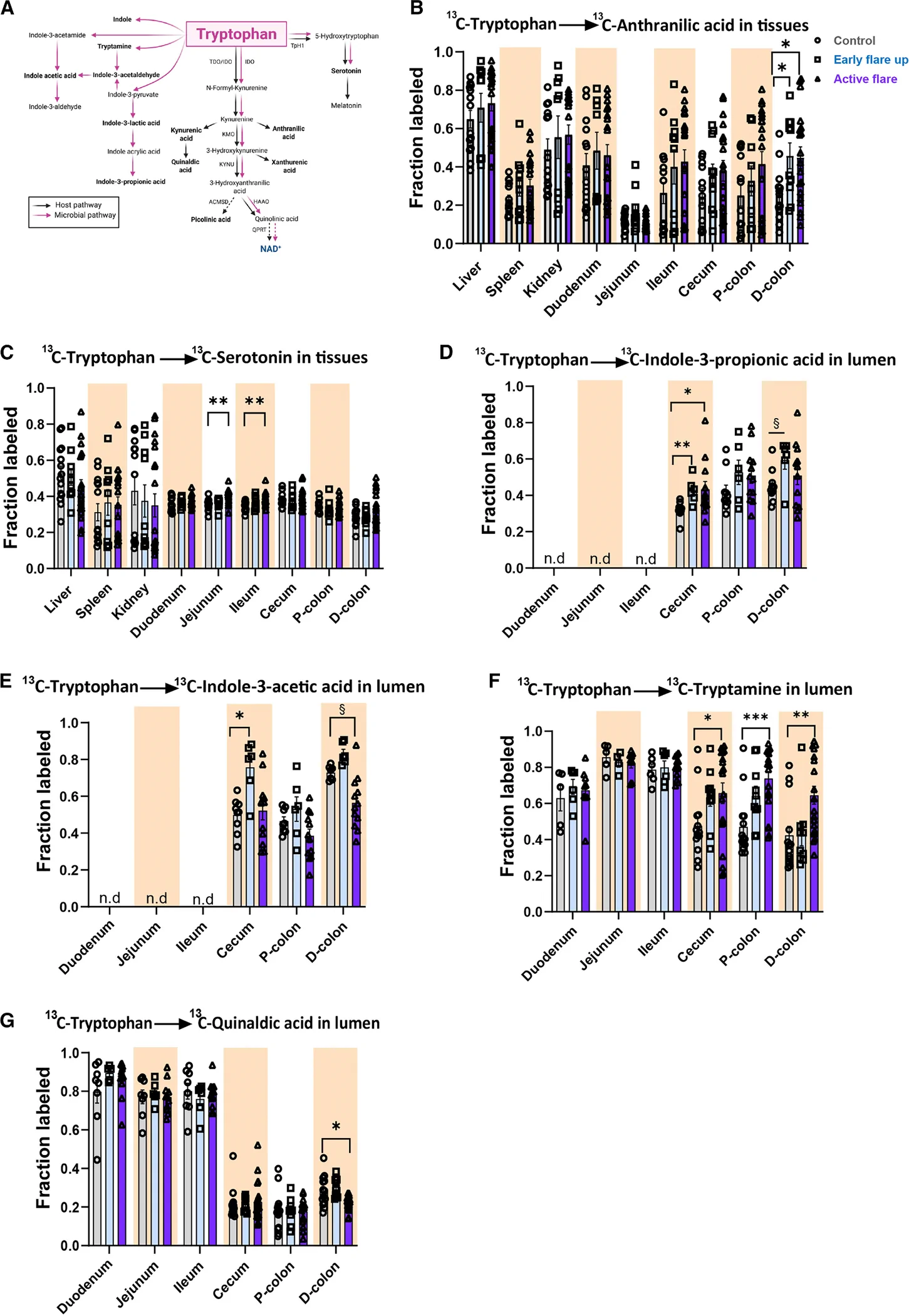
Acute colitis alters tryptophan-dependent pathways in the host tissues and gut microbes (A) Overview of tryptophan metabolism in host tissues and gut microbiota. Metabolites shown in bold are compounds measured in the study. (B–G) The fractional labeling of (^13^C_11_-tryptophan) over 20-h intravenous infusion (as in Figure 2), measured by LC-MS in different tissues for (B) anthranilic acid, (C) serotonin; and luminal regions of the gastrointestinal tract: (D) indole-propionic acid, (E) indole-acetic acid, (F) tryptamine, and (G) quinaldic acid. Data are presented as mean ± SEM (*n* = 10–20). Statistical significance was determined by Kruskal-Wallis test with post hoc Dunn’s test for comparisons among more than two groups. NS, not significant, § < 0.1, *p < 0.05, **p < 0.01, ***p < 0.001, and ****p < 0.0001. (A) was created with BioRender.com

We next investigated whether the degradation of Trp through the KP affects the production of metabolites from other Trp-dependent pathways, which may, in turn, alter the gut homeostasis. Apart from its role in NAD^+^ biosynthesis, the KP produces several bioactive metabolites from Kyn with distinct neuroactive and immunomodulatory properties (Figure 5A).^26^ Kynurenic acid (KynA) is produced from kynurenine (Kyn) by kynurenine aminotransferases (KAT I–IV), while anthranilic acid (AnthA) is generated from Kyn through the activity of kynureninase (KYNU). Similarly, xanthurenic acid (XanA) is formed from 3-hydroxykynurenine by KATs. In addition, picolinic acid (PicoA) is synthesized from 2-amino-3-carboxymuconate-6-semialdehyde (ACMS) through ACMS decarboxylase (ACMSD), which serves as a branch point that redirects the KP from production of QUIN and NAD^+^.^26^

In addition to the KP, Trp is also metabolized through the serotonin (5-hydroxytryptamine [5-HT]) pathway, in which serotonin serves as a key neurotransmitter involved in mood regulation, sleep, and GI motility.^26^ To address this, we measured the fractional labeling of Trp-derived metabolites using Trp (U-^13^C_11_) (M+11), as described in Figure 2, in host tissues and the gut lumen of DSS-treated and control mice at different stages of intestinal inflammation (Figure 5A).

Within host tissues, the fractional labeling of kynurenic acid (KynA) from Trp revealed a significant increase in the duodenum during the early flare-up phase; however, no differences in the fractional labeling were observed across other tissues during the active flare phase (Figure S5A). Labeled anthranilic acid (AnthA) was not altered in different tissues; however, we detected increased flux of Trp to AnthA in the colon in both early flare-up and active flare phases (Figure 5B). Flux of Trp to xanthurenic acid (XanA) and picolinic acid (PicoA) metabolites produced from the KP was not altered during the active phase of DSS treatment (Figures S5B and S5C). In addition to the KP- produced metabolites, the fraction of labeled serotonin was significantly increased in the jejunum and ileum during the active flare phase, suggesting enhanced flux of Trp to serotonin biosynthesis by enterochromaffin cells (ECs) in the gut lining.^38^ However, the flux of Trp to serotonin was not altered in other tissues, including colonic tissues (Figure 5C). We also quantified 5-methoxytryptophan (5-MTP) levels, a Trp-derived metabolite that may play a protective role against inflammation in preclinical studies.^39–41^ Acute intestinal inflammation had no detectable impact on the fractional labeling of 5-MTP in different tissues (Figure S5D). Additionally, the fractional labeling of quinaldic acid (QA), a Trp-derived metabolite produced from KynA, remained relatively stable in different tissues during different phases of intestinal inflammation (Figure S5E).

A growing body of evidence demonstrates that gut microbiota-derived indole and its derivatives produced in the intestine, including indole-3-acetic acid (IAA), indole-3-propionic acid (IPA), and indole-3-lactic acid (ILA), significantly influence intestinal barrier function and immune responses.^42–46^ To this end, we investigated Trp degradation into microbial indole and its derivatives. No difference in the fractional labeling of Trp to indole was observed across different luminal regions, except for a slight decrease in the cecal lumen during the active flare phase compared to controls (Figure S5F). The fractional labeling of ILA and indole-3-acetaldehyde (IAAld) derived from Trp was not significantly altered in different luminal regions (Figures S5G and S5H). The fractional labeling of IPA significantly increased in the cecum during both phases of intestinal inflammation; in addition, a slight increase was also observed in the D-colon during the early flare-up phase (Figure 5D). Microbial synthesis of (IAA) from Trp was reduced by approximately 15% (*p* = 0.0754) in the D-colon during the active flare phase, while it increased in the cecal lumen during the early flare-up phase (Figure 5E).

Tryptamine is a key Trp-derived metabolite that plays a dual role in inflammation, acting protectively under homeostasis, but potentially exacerbating inflammation during dysbiosis.^47–49^ We observed increased flux of Trp to tryptamine in the cecal and colon lumen during the active flare phase of inflammation, while flux in the small intestine remained unchanged, implying moderate effects of colitis on upper GI tract tryptamine production (Figure 5F).

Unlike host tissues, our data showed no alterations in the production of serotonin across diseased conditions in the intestinal lumen (Figure S5I). However, flux from Trp to luminal KynA increased in the ileum lumen during the active flare phase (Figure S5J). In addition, the fractional labeling of QA was reduced in the D-colon during the active flare phase, reflecting altered flux from Trp to QA during intestinal inflammation (Figure 5G). These data suggest that the systemic pool of Trp reaching the colon is utilized by the gut microbiota for metabolite production, with the generation of specific Trp-derived metabolites varying during the active phase of colitis. This is likely to reflect adaptive host-microbial responses aimed at maintaining gut homeostasis under inflammatory stress.

### Inflammation rewires tissue NAD^+^ metabolism through salvage pathway activation

**Figure 6.**
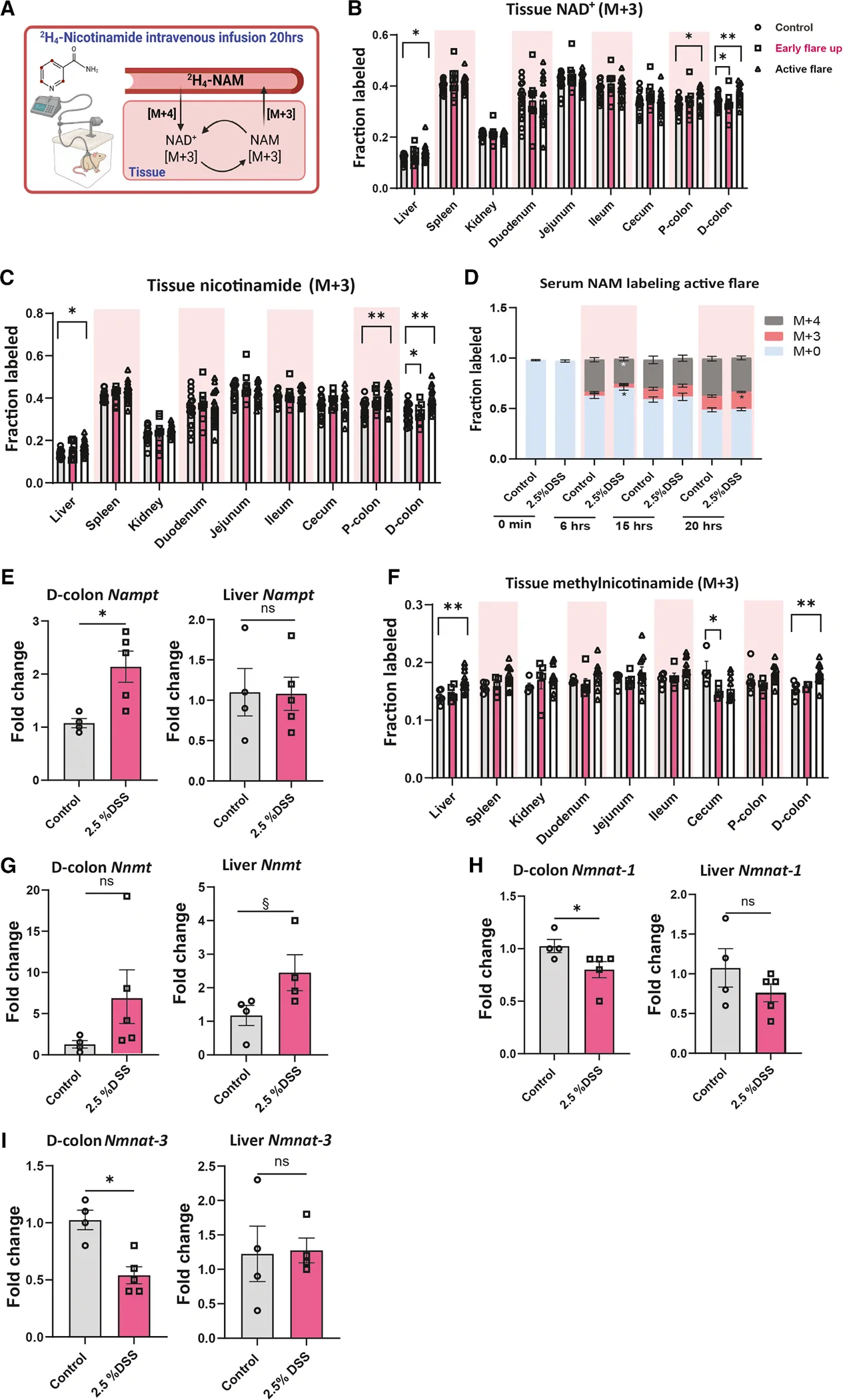
Inflammation enhances NAD^+^ production from nicotinamide via the salvage pathway in a tissue-specific manner (A) Overview schematic of experimental setup for intravenous infusion of deuterium-labeled nicotinamide (2,4,5,6-^2^H_4_-NAM) into pre-catheterized male C57BL/6 mice (11–12 weeks old) to assess NAD^+^ flux from nicotinamide (NAM) via the salvage pathway in host tissues. (B and C) Tissue fractional labeling following 20-h intravenous infusion of (B) NAD^+^ and (C) nicotinamide. (D) Fractional labeling of nicotinamide in the serum post 20-h intravenous infusion during the flare phase of intestinal inflammation (M+0: unlabeled NAM, M+3: recycled NAM, M+4: infusate NAM). (E) RT-qPCR analysis of mRNA expression normalized to *Tbp* in distal colon and liver tissues during the active flare phase (days 8 and 11) of nicotinamide phosphoribosyltransferase (*NAMPT*) compared to control. (F–I) Fractional labeling of methylnicotinamide in host tissues. RT-qPCR analysis of mRNA expression normalized to *Tbp* in distal colon and liver tissues during the active flare phase (days 8 and 11) compared to control for (G) nicotinamide N-methyltransferase (*NNMT*), (H) nicotinamide mononucleotide adenylyltransferase 1 (*NMNAT1*; nuclear), and (I) nicotinamide mononucleotide adenylyltransferase 3 (*NMNAT3*; mitochondrial). Data are presented as mean ± SEM, in (B)–(D) and (F), *n* = 8–18; in (E), (G), and (H), *n* = 4–5. Statistical significance was determined by Kruskal-Wallis test with post hoc Dunn’s test for comparisons among more than two groups and Mann-Whitney U test for two-group comparisons. NS, not significant, § < 0.1, *p < 0.05, **p < 0.01, ***p < 0.001, and ****p < 0.0001. (A) was created with BioRender.com

The salvage pathway is the major route of NAD^+^ production, balancing its continual consumption by NAD^+^ -consuming enzymes and maintaining cellular NAD^+^ levels by recycling NAM back to NAD^+^ via the rate-limiting enzyme nicotinamide phosphoribosyltransferese (NAMPT). This pathway is significant for both normal physiological cellular function and disease states, including IBD, where chronic inflammation imposes a high metabolic demand.^50–53^ To investigate whether the flux from NAM via the salvage pathway to NAD^+^ was impacted by acute colitis, we infused (2,4,5,6-^2^H) NAM (M+4) at a constant rate of 0.1 nmol/g body weight/min in DSS-treated and control mice at different phases of intestinal inflammation (Figure 6A). The deuterated NAM (M+4) used in the experiment has a deuterium atom at the redox-active (4-^2^H) site.^5,54^ This labeling remains with free NAM, but is lost once incorporated into NAD^+^. As a result, the tracer NAM (M+4) forms NAD^+^ (M+3), and NAD^+^ breakdown releases NAM (M+3), which can be recycled back to synthesize NAD^+^ or secreted into the circulation.^5,54^ The flux from NAM (M+4) to NAD^+^ (M+3) increased in the inflamed colon and liver during the active flare phase, along with elevated levels of recycled NAM (M+3), indicating higher NAD^+^ turnover in those tissues (Figures 6B and 6C). Circulating NAM (M+3) increased significantly during the active flare phase, but not during the early flare-up compared to control, reflecting increased NAD^+^ turnover during intestinal inflammation (Figures 6D and S6A).

We noted a significant 2-fold upregulation of *Nampt* expression in the colon (*p* = 0.0238) but not in the liver, indicating increased salvage pathway activity at the site of inflammation during the active flare phase (Figure 6E). Additionally, we quantified methylnicotinamide (MeNAM), a methylated product of NAM catalyzed by the nicotinamide N-methyltransferase (NNMT),^55^ and observed increased fractional labeling of MeNAM in the liver and colon during the active flare phase without a corresponding accumulation of the downstream metabolites N-Me-4PY and N-Me-6PY (Figures 6F, S6B, and S6C). This suggests enhanced NAM methylation, potentially to prevent excess NAM accumulation, which can inhibit sirtuins.^56,57^ *Nnmt* expression exhibited a trend toward upregulation in the liver and colon during the active flare phase; however, this was not statistically significant (*p* = 0.057 and *p* = 0.11, respectively) (Figure 6G). We observed a significant downregulation of nuclear *Nmnat-1* and mitochondrial *Nmnat-3* in the colon, but not in the liver (Figures 6H and 6I), whereas the expression of cytoplasmic *Nmnat-2* remained unchanged in both tissues during the active flare phase compared to controls (Figure S6D). These observations suggest that the salvage pathway is selectively activated in metabolically stressed tissues, including the liver and colon during acute colitis, reflecting local and systemic adaptation. This likely represents a coordinated adaptive response to depleted NAD^+^ levels in the colon during colitis, boosting the use of NAM for NAD^+^ synthesis, suggesting a coordinated metabolic activation to support NAD^+^ replenishment under inflammatory stress.

### The cycling of NAD^+^ precursors between the host tissues and the gut microbiota persists with region-specific alterations during acute colitis

Given the role of NAD^+^ precursor exchange between the host tissues and gut microbiota, we next examined whether acute DSS-induced colitis disrupts the interconversion of NAD^+^ precursors between the host tissues and the gut microbiota.

**Figure 7.**
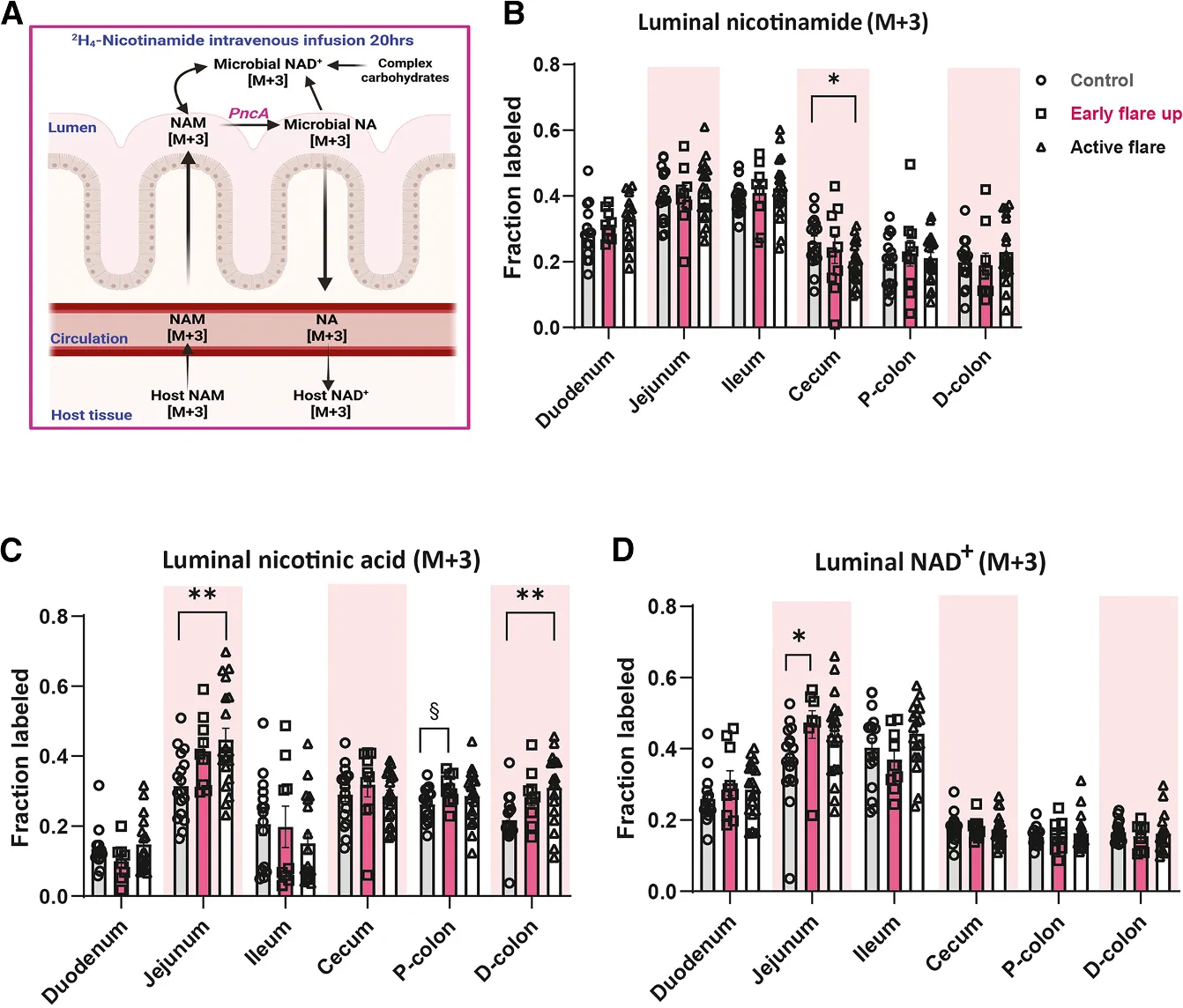
Acute intestinal inflammation does not compromise the metabolic cycling of NAD^+^ precursors between host tissues and the gut microbiota Schematic of the (A) metabolic exchange of the NAD^+^ precursors nicotinamide (NAM) and nicotinic acid (NA) between host tissues and the gut lumen. Host-derived NAM enters the gut lumen and contributes to microbial NAD^+^ either through conversion to NA and subsequently to NAD^+^, which supports both host and microbial NAD^+^ biosynthesis, or is directly converted to NAD^+^. Complex carbohydrates also contribute to microbial NAD^+^. LC-MS measurements of luminal microbial metabolites in the NAD^+^ salvage pathway after 20-h intravenous infusion of the nicotinamide tracer (described in Figure 6); (B) luminal nicotinamide, (C) luminal NA, and (D) luminal NAD^+^. Data are presented as mean ± SEM (*n* = 8–18). Statistical significance was determined by Kruskal-Wallis test with post hoc Dunn’s test for comparisons among more than two groups. § < 0.1, *p < 0.05, **p < 0.01, ***p < 0.001, and ****p < 0.0001. (A) was created with BioRender.com.

Production of luminal NAD^+^ can occur via multiple routes: (1) host-derived NAM to microbial NA to NAD^+^, a major route, (2) host-derived NAM to NAD^+^, or (3) through complex carbohydrates that support *de novo* synthesis of NAD^+ 7^ (Figure 7A). We quantified the labeling patterns of luminal NAM, NA, and NAD^+^ from infused NAM (M+4) using the tracer described in Figure 6A. The host-derived NAM (M+3) was detected in the lumen throughout the GI tract during intestinal inflammation, although it slightly decreased in the cecal lumen during the active flare phase compared to control (Figure 7B). Labeled microbial NA (M+3), generated from NAM (M+3), increased in the lumen of the jejunum and D-colon in the active flare phase, suggesting localized enhancement of NAM-to-NA conversion (Figure 7C). NAM (M+4) was not detected in the luminal samples, supporting the notion that luminal NAM (M+3) is primarily derived from host tissues into the gut lumen. Luminal NAD^+^ (M+3) production from the shared precursors NAM and NA was comparable in DSS-treated and control mice in both phases, with a slight increase in the jejunum during the early flare-up phase (Figure 7D). Labeled NA (M+3) was detected in the small intestine and colon, indicating the uptake of microbial NA (M+3) by the host tissues and reflecting the dynamic interactions between the gut microbiota and host tissues (Figure S7A). The gene expression of nicotinate phosphoribosyltransferase (*Naprt*) was comparable between DSS-treated mice and controls in colonic tissues, supporting the observed uptake of microbial NA by the host tissues (Figure S7B). Together, these findings suggest that the host-microbiota NAD^+^ precursor cycling is maintained and locally modulated during intestinal inflammation, highlighting the resilience of the metabolic interactions during acute colitis.

### Trp catabolism during acute colitis induction leads to a systemic metabolic rewiring to replenish NAD^+^

**Figure 8.**
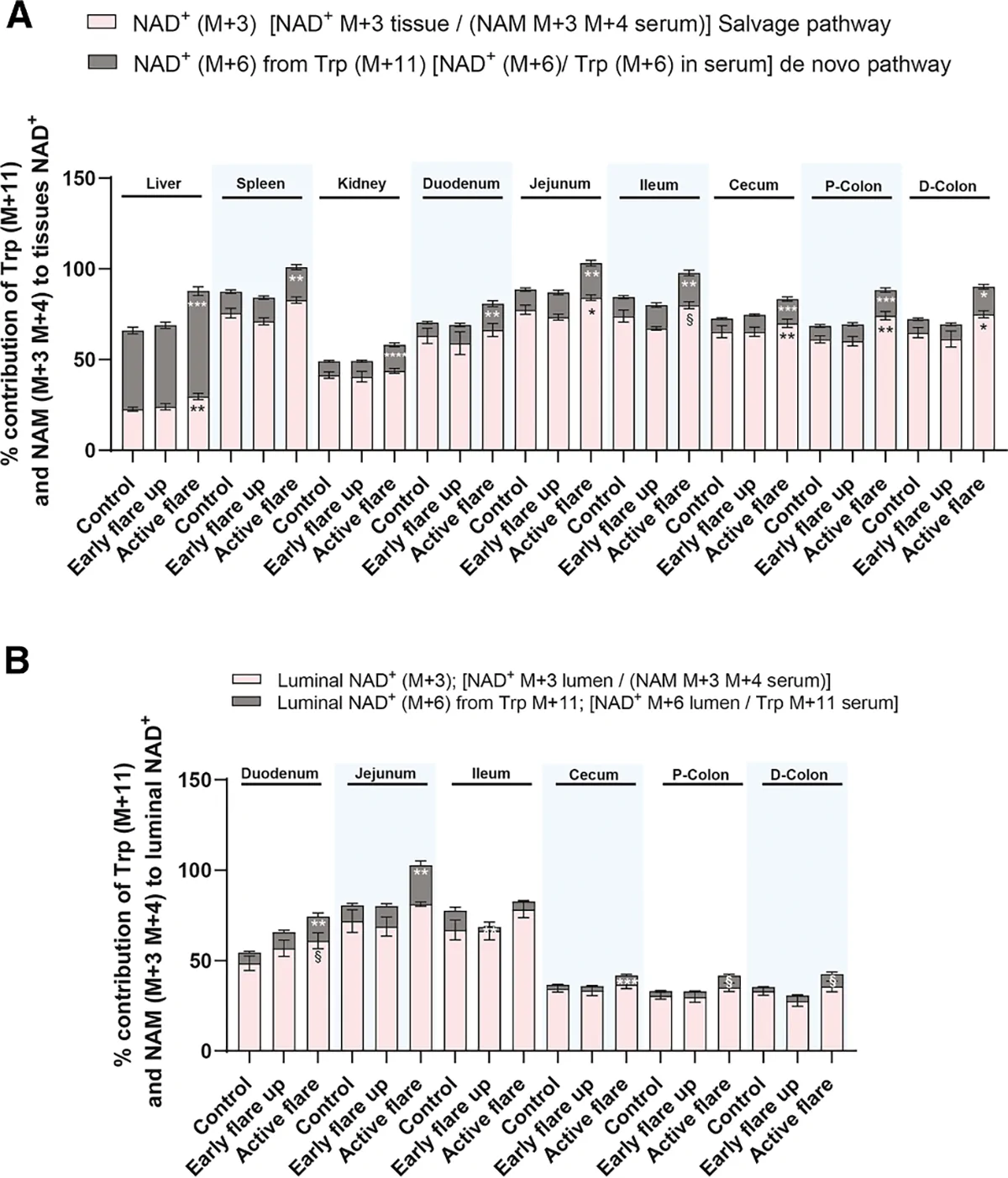
Acute colitis triggers systemic metabolic adaptation to restore NAD^+^ levels through activation of the salvage pathway Percent contributions of tryptophan and nicotinamide to (A) host NAD^+^ levels in different tissues and (B) luminal NAD^+^ across different phases of intestinal inflammation. In (A) and (B), the contribution of nicotinamide (M+4) to NAD^+^ (M+3) was calculated as [fraction of labeled NAD^+^ (M+3) / the sum of (NAM+3 and NAM+4) in serum]. Tryptophan (M+11) contribution to tissue NAD^+^ (M+6) was quantified as [fraction of labeled NAD^+^ (M+6)/fraction of labeled Trp (M+11) in the serum], following 20-h intravenous infusion of the labeled NAD^+^ precursors (depicted in Figures 2 and 6). Asterisks indicate statistical significance compared with the corresponding control group within each tissue or intestinal luminal regions. Data are presented as mean ± SEM. Statistical significance was determined by Kruskal-Wallis test with post hoc Dunn’s test for comparisons among more than two groups. § < 0.1, *p < 0.05, **p < 0.01, ***p < 0.001, and ****p < 0.0001.

Given the observed decline in NAD^+^ levels within colonic tissues and luminal contents, alongside compensatory increases in NAD^+^ production across other tissues during the acute colitis active flare phase, we next sought to determine the relative contributions of Trp and NAM to NAD^+^ pools in host tissues and the intestinal lumen. The analysis revealed that the liver relies on Trp for NAD^+^ production. During the active flare phase, a significant increase in Trp-derived NAD^+^ was observed in the spleen, kidney, small intestine, and colon. However, these tissues primarily depend on the salvage pathway for NAD^+^ production—using NAM directly or indirectly through recycled NAM (M+6) generated from Trp-derived NAD^+^. In colonic tissues, this recycling provides an alternative route to bypass the blockade in *de novo* NAD^+^ synthesis from Trp^5,25,54^ (Figure 8A). The analysis of luminal NAD^+^ indicated that under physiological conditions, the salvage pathway from NAM is the primary source of luminal NAD^+^. However, during the active flare phase, there was a significant increase in the contribution of Trp to luminal NAD^+^ in the duodenum, jejunum, and cecum, accompanied by a significant decrease in NAD^+^ production from Trp in the ileum lumen during the early flare-up phase. Additionally, a modest upward trend in Trp-derived luminal NAD^+^ was observed in the colon lumen (Figure 8B). The expression profiling of NAD^+^-consuming enzymes indicated no significant differences between the DSS-treated and control groups, except for CD38, which showed a modest increase, though not statistically significant, in both the colon and liver during the active flare phase (Figure S8A).

Collectively, these findings demonstrate that acute intestinal inflammation reshapes NAD^+^ metabolism in both host tissues and the gut microbiota, with coordinated changes in the *de novo* and salvage pathways as part of the NAD^+^ metabolic response to acute colitis.

## DISCUSSION

Although previous studies have reported alterations in NAD^+^ levels in IBD, it remains unclear whether the imbalance is due to changes in NAD^+^ biosynthesis, consumption, or both.^8,9,58,59^ Dysregulated NAD^+^ levels disrupt cellular metabolism and homeostasis, driving a cascade of physiological dysfunction. Impaired NAD^+^ availability can disturb energy metabolism, hinder cellular repair mechanisms, and escalate oxidative stress, which may exacerbate inflammatory processes in IBD.^60–63^

In acute DSS-induced colitis, we observed a pronounced reduction in NAD^+^ levels in the inflamed colon and adjacent lumen. This decline is driven, at least in part, by impaired *de novo* NAD^+^ synthesis from Trp in the inflamed mucosa due to a metabolic bottleneck at *Qprt*,^25^ resulting in insufficient NAD^+^ production to meet increased metabolic demands. In compensation, acute colitis induces extensive metabolic reprogramming, with distinct NAD^+^ biosynthetic pathways activated across tissues according to their specific metabolic needs during inflammatory stress. In host tissues, activation of the NAM-dependent salvage pathway provides a rapid, tissue-specific metabolic response to replenish depleted NAD^+^ levels under inflammatory stress. In parallel, the gut microbiota sustains NAD^+^ production primarily through the salvage pathway, while *de novo* synthesis contributes to microbially derived NAD^+^ during acute DSS-induced inflammation (graphical abstract).

The increased flux of Trp into NAD^+^ across multiple tissues underscores a systemic adaptive response mediated by activation of the KP, likely driven by pro-inflammatory cytokine signaling and immune activation.^64–66^ Although *de novo* synthesis remains obstructed at *Qprt* in the colon, Trp continues to serve as a key precursor for NAD^+^ production in the liver, where recycling of NAD^+^ to NAM enables redistribution of NAM to the colon to sustain NAD^+^ regeneration through the salvage pathway. The liver and colon thus exhibit distinct yet complementary NAD^+^ biosynthetic responses to inflammation, with enhanced flux through the salvage pathway in both tissues supporting the maintenance of NAD^+^ homeostasis under acute inflammatory conditions.

The enhancement of both the KP and salvage pathway during acute colitis suggests a compensatory mechanism to regenerate depleted NAD^+^ levels in the inflamed mucosa, thereby meeting the increased NAD^+^ demand associated with inflammation. This aligns with previous studies showing that inflammatory cytokines can drive metabolic changes to support immune responses, genomic stability, and tissue repair.^67,68^

An important aspect of our findings is the role of the gut microbiome in regulating NAD^+^ metabolism during inflammation. The increased flux of Trp to NAD^+^ in luminal regions suggests that microbial metabolism substantially contributes to local NAD^+^ production under inflammatory conditions. Additionally, the host-microbiota NAD^+^ precursor cycling appears to be preserved during acute intestinal inflammation, but is subject to region-specific modulation, supporting a model in which adaptive, region-specific regulation of NAD^+^ metabolism helps sustain NAD^+^ homeostasis. However, while these data are consistent with the contribution of microbial NAM-to-NA-to-NAD^+^ precursor cycling, the precise quantitative contribution of microbial versus host pathways cannot be resolved with the current tracer approach. This highlights the dynamic interplay between host and microbial metabolism, where microbial activity can shape host tissue responses and influence energy and immune balance during inflammatory stress.^69,70^ Moreover, given the microbiome’s capacity to synthesize NAD^+^ from precursors such as Trp and NAM,^7^ microbially derived NAD^+^ and related metabolites likely support the systemic NAD^+^ pool, reinforcing host metabolic resilience and modulating inflammatory responses in the context of IBD.^71,72^

The observed increase in labeled Trp in the colonic lumen may reflect alterations in bacterial Trp catabolism during acute inflammation. Our data reveal a shift in certain Trp-metabolizing bacteria, which may alter the host-microbiome dynamics and impact gut homeostasis. Future work should further investigate the potential role of the altered gut bacteria involved in Trp metabolism, deciphering how they affect systemic metabolism and impact disease progression in chronic colitis.

Given the importance of NAD^+^ in immune function and tissue repair,^73^ modulating NAD^+^ metabolism may represent a promising therapeutic strategy for IBD. Our multi-omics approach provides a comprehensive overview of NAD^+^ metabolism in IBD, revealing important insights into tissue-specific metabolic responses and the role of the gut microbiota. Targeting key NAD^+^ biosynthesis pathways, particularly the salvage pathway, could help restore NAD^+^ homeostasis and mitigate inflammation in the gut.^53^ In line with this concept, we are currently evaluating an ileocolonic-release formulation of oral NAM for the treatment of mild to moderate UC (Ornatus 1, NCT06488625). Future studies should aim to dissect the contributions of individual microbial species to NAD^+^ production and explore how these interactions affect host health in other inflammatory diseases.

In summary, our study reveals that acute colitis induces a complex metabolic response, where distinct NAD^+^ biosynthesis pathways are activated in different tissues to meet the metabolic demands of inflammation. By integrating isotope tracing and multi-omics techniques, we have provided new insights into the role of NAD^+^ metabolism in IBD and highlighted potential therapeutic targets for restoring metabolic homeostasis in inflammatory diseases.

## RESOURCE AVAILABILITY

### Lead contact

Further information and requests for resources and reagents should be directed to the lead contact, Melanie R. McReynolds (mcreynolds@psu.edu).

### Materials availability

The study did not generate new materials.

### Data and code availability

- All metabolomics and isotope-tracing data reported have been deposited at MassIVE (mass spectrometry interactive virtual environment) and are publicly available as of the date of publication. The repository can be accessed at ftp://MSV000102197@massive-ftp.ucsd.edu. Accession numbers are listed on the key resources table.
- Custom R scripts and computational workflows used for metagenomics downstream analysis are publicly available at https://gitlab.com/mcreynoldslab-group/McReynoldsLab-project/-/releases/Metagenomics_Analysis.
- Any additional information required to reanalyze the data reported in this paper is available from the lead contact upon request.

## STAR★METHODS

### EXPERIMENTAL MODEL AND STUDY PARTICIPANT DETAILS

#### Mice

11–12-weeks-old male C57BL/6 (WT) mice pre-catheterized on the right jugular vein were purchased from Charles River Laboratories (Wilmington, MA). Animals were single-housed in a temperature-controlled facility and maintained on 12-hour-light-dark cycle (7AM-7PM). All mice were given *ad libitum* access to a normal chow diet (catalog# 5053, LabDiet) and acclimated for at least 7 days before experimental use. Animal studies were conducted at Pennsylvania State University and approved by the Institutional Animal Care and Use Committee (PROTO202202188).

### METHOD DETAILS

#### Acute DSS colitis induction in mice

Mice were randomly assigned into 2 groups (n = 8–20 group). The control group received normal drinking water without DSS, and the treated group was administered dextran sulfate sodium (DSS) (MW 36–50kDa, MP Biomedicals, Solon, OH) at a concentration of 2.5% (w/v) in drinking water for 5 days.^17,18^ Following the initial treatment period (5 days), both groups received normal drinking water until the end of the experiment on day 11. The early flare up phase is characterized by mild colitis occurring on days 4–5, and the active flare phase spans from days 7–11, in which the colon is fully inflamed.^19–21^ During the experiment, mice were weighed daily, and fecal and serum samples were collected on days (1,3,5,8,11) and stored at −80°C. At the end of the experiment, mice were sacrificed by cervical dislocation, tissues and luminal samples were collected and snap frozen in liquid nitrogen.

#### Clinical assessment of colitis severity

The severity of colitis was assessed by evaluating the following parameters daily: weight loss (0 points = no weight loss or gain, 1 point = 1–5% weight loss, 2 points = 6–10% weight loss, 3 points = 11–19% weight loss, 4 points = 20–25% weight loss, 5 points = >25% weight loss); stool consistency (0 points = normal and firm, 1 point = very slight change, 2 points = slight change and soft, 3 points = moderate change, 4 points = noticeable change, diarrhea, 5 points = severe change, runny diarrhea; bleeding stool (0 points = normal, 1 point = redness of perianal region, 2 points = slightly blood-streaked stool, 3 points = blood-streaked stool, 4 points = marked blood contamination, 5 points = bloody stool); posture (0 points = normal, 1 points = very slight change, 2 points = slightly curved, 3 points = moderate change, 4 points = strongly curved, 5 points = severe change and consistently curved); activity (0 point = normal active, 1 point = very slight change in activity, 2 points = reduced movement and clinging onto the cage, 3 points = moderate change, 4 points = noticeable change and rarely clinging onto the cage, 5 points = severe change, sitting still and no movement); fur (0 points = normal, 1 point = very slight change, 2 points = slightly dirty and scruffy, 3 points = moderate change, 4 points = noticeable change, dirty, and scruffy, 5 points = severe change, very dirty, dull, and scruffy). The DAI was calculated by simple summation of the individual scores for the measured parameter.^18,78^ DAI score = wright loss compared to initial weight score+ stool consistency score+ fecal bleeding score+ posture score+ activity score+ fur score observed daily.

#### Histological analysis of disease activity

Postmortem, colonic tissues were excised and cut open longitudinally. The colon was rolled up as Swiss rolls from the distal to the proximal part and fixed in 10% formalin. Paraffin sections were cut and stained with hematoxylin and eosin (H&E). Histological scoring displays the combined score of inflammatory cell infiltration and tissue damage as described elsewhere and was performed in a blinded fashion, as described previously^79^

#### Murine immunohistochemistry

Formalin-fixed paraffin-embedded colon section slides were deparaffinized in Xylene-substitute (Carl Roth GmbH + Co. KG, Karlsruhe, Germany) and rehydrated in ethanol (Th. Geyer GmbH & Co. KG, Hamburg, Germany). Antigen retrieval was carried out by heating the slides in citrate buffer (10mM, PH 6.0, prepared in the laboratory) for 20 min. The sections were submerged in 3% hydrogen peroxide (Sigma-Aldrich, Merck KGaA, Darmstadt, Germany) for 10 min to block endogenous peroxidases, and nonspecific binding was blocked using 5% BSA (Carl Roth GmbH + Co. KG, Karlsruhe, Germany) in PBS (Life Technologies GmbH, Darmstadt, Germany) for 1 h. Tissues were incubated with the primary antibody Qprt (1:75, Biorbyt, orb317756) at 4°C overnight followed by a 45-min incubation with a biotinylated secondary antibody (goat anti-rabbit IgG ready-to-use, Abcam, ab64256). Signal detection was performed using the Vectastain ABC Kit (Vector Laboratories, Peterborough, UK) according to the manufacturer’s instructions. Color development was carried out using the DAB Peroxidase Substate Kit (Vector Laboratories, Peterborough, UK). Tissue sections were then counterstained by hematoxylin (Sigma-Aldrich, Merck KGaA, Darmstadt, Germany), dehydrated in ethanol (Th. Geyer GmbH & Co. KG, Hamburg, Germany) and mounted with ROTI-Histokitt mounting medium (Carl Roth GmbH + Co. KG, Karlsruhe, Germany).

To quantify immunohistochemical (IHC) staining intensity, ten images per Swiss roll were acquired using Axio Observer A1 brightfield microscope (Zeiss, Germany) at 40*x* magnification with ZEN software under identical imaging conditions (exposure time, white balance, and color calibration). Images were analyzed in ImageJ version 1.54 g using the IHC Toolbox plugin (https://imagej.net/ij/plugins/ihc-toolbox/) with the H-DAB model to isolate the brown signal corresponding to the secondary antibody. The resulting images were converted to 8-bit grayscale and thresholded (0–120) prior to quantitative analysis to determine the stained area and mean gray value. Staining intensity was calculated as the product of mean gray value and stained area. For each animal, values from technical replicates were averaged to obtain a single mean value.

#### Enzyme-linked immunosorbent assay (ELISA)

Quantification of fecal Lipocalin-2 was used to assess intestinal inflammation. Briefly, fresh or frozen fecal samples from control and DSS-treated (active flare phase) mice were reconstituted in PBS containing 0.1% Tween 20 (100mg/mL) and vortexed for 20 min to create a homogenous suspension. The samples were then centrifuged at 12,000 rpm for 10 min at 4°C. Clear supernatants were collected and stored at −20°C until analysis. Lcn-2 levels in the supernatants were measured using ELISA kit purchased from (R&D systems DY1857, Minneapolis, MN, USA) following manufacturer’s instructions.

#### RNA isolation and quantitative real-time PCR (qRT-PCR)

Total RNA was extracted from liver and colon tissues using TRIzol reagent (Thermo Fisher Scientific, Carlsbad, CA, USA) per the manufacturer’s protocol. The purified RNA 1.0 μg was reverse transcribed into cDNA using qScript cDNA synthesis kit (Quanta Biosciences, Beverly, MA, USA). Quantitative RT-PCR was performed using the PerfeCTa qPCR FastMix, UNG, Low ROX (Quanta Biosciences, Beverly, MA, USA) in a 20 μl reaction mixture containing cDNA and TaqMan probes (Thermo Fisher Scientific) per the manufacturer’s instructions for TaqMan assays. The cycling parameters were 95.0 C for 3 min, followed by 40 cycles of 95.0 C for 15 s, and 60C for 1 min. Relative quantification of each gene was calculated using 2^−ΔΔCt^, and normalized to TBP expression to yield a fold-change. A list of the primers used in the study is provided in supplement Table S1.

#### Intravenous infusion of mice

*In vivo* infusion of stable isotope labeled NAD precursors, [2,4,5,6-^2^H]-NAM and [U-^13^C_11_]-Trp (Cambridge Isotope Laboratories, Andover, MA, USA), were infused separately in control and DSS-treated mice at different stages of intestinal inflammation (early flare up phase = day 4–5 post colitis induction; active flare phase = day 7–8, and day 10–11) for 20 h to achieve steady state NAD^+^ labeling from labeled precursors in different tissues. The mouse infusion setup included a tether and swivel system (Instech Laboratories, Plymouth Meeting, PA) to allow free movement of the mouse in the cage with bedding materials and access to food and hydrogel water (Clear H_2_O, Portland, ME). Isotope labeled NAD^+^ precursors were prepared as a solution in 0.9% NaCl (50mM for [U-^13^C_11_]-Trp, and 4mM for [2,4,5,6-^2^H]-NAM) and infused via the catheter at a constant rate of 0.5 μL per 20 g body weight per min. Blood samples (~20 μL) were collected via tail bleeding using microvette CB 300 CAT blood collection tubes (ref. 16.440.100, SARSTEDT AG& Co.KG, Nümbrecht, Germany) at different time points (0min, 15min, 30min, 1hr, 2hr, 6hr, 15hr, and 20hr) and centrifuged at 16,000 g for 25 min at 4°C to separate serum. At the end of the infusion, mice were euthanized by cervical dislocation. Tissues and luminal samples were dissected and separated and immediately clamped with a pre-cooled Wollenberger clamp in foil and stored in liquid nitrogen. Tissues, serum, lumen, fecal samples were kept at −80°C prior metabolites extraction for mass spectrometry analysis.^54^

#### Metabolite extraction from serum, tissues, and lumen

To preserve redox-sensitive metabolites and minimize artifactual metabolite interconversion, all samples were maintained under cold conditions throughout processing and extracted immediately following a single freeze–thaw cycle. Serum samples were thawed on ice and immediately extracted using pre-chilled (−20°C) 100% methanol at a ratio of 65 μL solvent per 5 μL serum. Samples were vortexed for 15 s, rapidly quenched on dry ice for 10 min, and centrifuged at 16,000 × g for 25 min at 4°C. The resulting supernatants were transferred to MS vials (Thermo Scientific, Rockwood, TN, USA) and maintained at 4°C prior to LC–MS analysis.

Tissues, lumen, and fecal samples (~20 mg) were cryogenically homogenized in liquid nitrogen using a cryomill (Retsch) at 25 Hz for 45 s prior to extraction with ice-cold 40:40:20 acetonitrile:methanol:water at a ratio of 40 μL solvent per mg tissue. Samples were vortexed for 10 s, incubated on ice for 10 min to facilitate protein precipitation and metabolite stabilization, and centrifuged at 16,000 × g for 30 min at 4°C. Supernatants were transferred to fresh 2 mL tubes and subjected to a second centrifugation step at 16,000 × g for 30 min to remove residual particulates. The upper 50 μL of clarified extract was transferred for LC–MS analysis. These extraction procedures were optimized for NAD^+^ and other redox-labile metabolites based on prior studies^5,7,54,80,81^ Across all sample types, rapid quenching, minimized handling time, cold solvent extraction, and cryogenic processing were implemented to reduce enzymatic activity and preserve metabolite integrity during sample preparation.

#### LC-MS metabolite measurement and isotope tracing analysis

Metabolite extracts were analyzed within 24 h of extraction using liquid chromatography coupled to high-resolution mass spectrometry (LC–MS). Chromatographic separation was performed using hydrophilic interaction chromatography (HILIC) on an XBridge BEH Amide column (2.1 × 150 mm, 3.5 μm particle size; Waters, Milford, MA) coupled to an Orbitrap Exploris 240 mass spectrometer (Thermo Scientific) as previously described.^82^

Solvent A consisted of 95:5 H_2_O:acetonitrile containing 20 mM ammonium acetate and 20 mM ammonium hydroxide, while solvent B consisted of 90:10 acetonitrile:H_2_O containing 20 mM ammonium acetate and 20 mM ammonium hydroxide. The gradient was as follows: 0–2 min, 90% B; 3–7 min, 75% B; 8–9 min, 70% B; 10–12 min, 50% B; 13–14 min, 25% B; 16–20 min, 0% B; and re-equilibrated to 90% B from 21 to 25 min. The flow rate was maintained at 150 μL/min with a column temperature of 25°C, autosampler temperature of 5°C, and injection volume of 5 μL. The mass spectrometer was operated in both positive and negative ionization modes with a resolving power of 180,000 at m/z 200, automatic gain control (AGC) target of 3 × 10^6^, maximum injection time of 30 ms, and scan range of m/z 70–1000. Instrument calibration was performed prior to data acquisition, and metabolite measurements were acquired with a mass accuracy of <10 ppm.

Metabolite identities were established using MS/MS-confirmed spectral matching against commercial spectral libraries and in-house authentic standards, which together comprise our curated list of validated NAD^+^-related metabolites (“knowns”). Supplementary Table S2 provides metabolite-specific information including retention times, mass-to-charge ratios (m/z), and ionization modes. MS/MS spectra were used for metabolite identification and validation, while quantitative isotope tracing analyses were performed using high-resolution MS1 acquisition to preserve intact isotopologue distributions and enable accurate assessment of isotope enrichment patterns.

For isotope tracing studies, isotopologue distributions were corrected for natural isotope abundance (^13^C and ^2^H) using AccuCor.^83^ Metabolite abundances were normalized to sample input (e.g., tissue weight or serum volume) and internal standards were available. Isotope-labeled internal standards were included for select NAD^+^-related metabolites to account for extraction and instrument variability. For metabolites lacking commercially available labeled standards, relative quantification was performed using consistent extraction procedures, chromatographic separation, and high-resolution MS detection across all experimental groups.

To ensure analytical reproducibility, blank samples were analyzed to monitor background signal and potential carryover, and samples were randomized prior to LC–MS acquisition to minimize run-order and batch-related effects. Raw LC/MS data were converted to mzML format using the command-line “msconvert” utility and analyzed using EL-MAVEN software version 12. The isotope tracing framework provided an internally controlled measure of pathway activity and metabolic flux, enabling robust relative comparisons of isotopic enrichment patterns across experimental conditions.

#### Metagenomic DNA extraction

Mice fecal samples from control and DSS-treated groups (flare phase) selected for metagenomic sequencing were extracted using the DNeasy PowerSoil Kit (Qiagen, Germantown, MD) following the standard protocol in the One Health Microbiome Center Co-Laboratory at Pennsylvania State University. Detailed protocol can be found at https://github.com/BisanzLab/OHMC_Colaboratory. Extraction blanks were included to assess environmental and reagent contamination. Briefly, fecal samples were weighed (~50 mg) and homogenized after bead beating for 5 min at 25 Hz using Qiagen Tissuelyzer III (Qiagen, Germantown, MD). Protein and solid particles were precipitated, and genome DNA was further purified on provided columns. Purified genomic DNA was eluted in nuclease-free H_2_O. Genomic DNA was quantified by spectrophotometry (Nanodrop One) and shipped to Novogene (Sacramento, CA) on dry ice for library preparation and sequencing via NovaSeq X with PE150 reads. Samples were sequenced with an 4.5 ± 1.9 Gbases of sequencing data (mean ± sd).

#### Metagenomic sequencing and data processing

Samples were processed by first using FastP to detect and remove adapters, remove polyG runs, and perform sliding window quality filtering.^76^ Next reads mapping to host DNA were removed using Kneaddata using provided reference assemblies.^84^ Sample normalization factors were determined using MicrobeCensus.^85^ Taxonomic abundances were determined using MetaPhlAn v4.0.6 including viruses and unclassified estimation. Gene family abundances and pathway abundances were determined using HUMAnN v3.9 against the uniref. 90 database.^75^ Taxonomic and Pathway abundances were normalized as the log2 of the reported abundance. Gene family abundances were normalized as RPKG (read per kilobase per genome equivalent as derived from MicrobeCensus). Alpha diversity metrics were determined using Vegan v2.6–10. Principal coordinates analysis was performed as implemented in Ape v5.8–1. Statistical analysis was performed log-transformed data and Welch’s *t* test. PerMANOVA was implemented using the adonis2 function of Vegan with 999 permutations. All *p*-values were corrected with Benjamini Hochberg False Discovery Rate unless otherwise indicated.

#### Integrative multi-omics network analysis of tryptophan metabolism

To identify microbial taxa capable of metabolizing tryptophan and its derivatives, we used an integrative knowledge-based multiomics integration approach using the OmicsNet 2.0 web interface.^74^Tryptophan-related metabolites captured through targeted metabolomics analysis and MetaPhlAn-derived species-abundances were used as an input for the network construction via logistic regression models trained on high-quality genome-scale metabolic models (GEMs). EMBL GEM repository were used to construct an initial metabolite–taxon interaction network, with confidence threshold of ≥0.7 and excluding metabolites without defined pathway annotations (e.g., KEGG or MetaCyc). The resulting global network was converted to a zero-order network to retain only species and metabolites that were part of the original input seed set.

#### Western blot analysis

Normalized tissue weights (~25–30mg) were collected from targeted tissues and lysed with RIPA buffer with 1% Halt Protease and Phosphatase Inhibitor Cocktail (ThermoFisher Scientific). Lysates were centrifuged at 16,000 g for 15 min at 4°C prior to quantification by BCA Protein Kit (ThermoFisher Scientific). 20 μg of sample per well were detected by gradient 7–14% SDS-PAGE and the protein bands were transferred into the 0.2 μm PVDF membranes (BioRad, 1704156). After incubation for 1 h with 5% blotting grade milk, the membranes were put with primary antibody (IDO1, IDO2, TDO2 and b-Actin antibody) at 4°C all night followed by incubation with HRP-conjugated secondary antibody for 1 h and the results were evaluated using electrogenerated chemiluminescence (BioRad, 170506).

### QUANTIFICATION AND STATISTICAL ANALYSIS

Statistical analysis was performed using GraphPad PRISM software version 10.4.0 (GraphPad Software, La Jolla, CA, USA). Unless otherwise stated, all data are presented as the mean ± SEM. Comparisons between two groups were performed using Mann-Whitney U test. For comparison between more than two groups, Kruskal-Wallis test followed by Dunn’s multiple comparisons was applied. Statistical significance was defined as (§ <0.1, *p < 0.05, **p < 0.01, ***p < 0.001, and ****p < 0.0001). Unless otherwise indicated, n represents the number of individual biological samples from two independent experiments. Statistical details for each experiment are provided in the corresponding figure legends.

## Supplementary Material

1

SUPPLEMENTAL INFORMATION

Supplemental information can be found online at https://doi.org/10.1016/j.celrep.2026.117730.

## Figures and Tables

**KEY RESOURCES TABLE:** 

REAGENT or RESOURCE	SOURCE	IDENTIFIER

Antibodies		

Indolamine2,3-dioxygenase 1 (IDO1) monoclonal Antibody	Thermo Fisher	CAT# 66528-1-IG; LOT: 10012243; RRID: AB_2881891
Indolamine2,3-dioxygenase 2 (IDO2) Polyclonal Antibody	Thermo Fisher	CAT# PA5-143950; LOT: 80392168; RRID: AB_3075164
Tryptophan 2,3-dioxygenase (TDO2) Polyclonal Antibody	Thermo Fisher	CAT# 15880-1-AP; LOT: 0015532; RRID:AB_2827610
beta Actin Polyclonal Antibody	Thermo Fisher	CAT# PA5-85271; LOT: 79422113; RRID:AB_2792414
Goat Anti-Rabbit IgG H&L (HRP)	Abcam	CAT# ab6721; LOT:1094269-5; RRID:AB_955447
Goat Anti-Mouse IgG H&L (HRP)	Abcam	CAT# ab205719; LOT: 1105770-1; RRID:AB_2755049
QPRT/QPRTase Rabbit Polyclonal Antibody	Biorbyt	RRID:AB_orb317756
Biotinylated Goat Anti-Rabbit IgG (H + L) (Ready to use)	Abcam	CAT# ab64256; RRID:AB_2661852

Chemicals, peptides, and recombinant proteins		

Dextran sulfate sodium (DSS)	MP Biomedicals	CAT# 160110
[U-^13^C] Tryptophan	Cambridge Isotope Laboratories	CAT# CLM-4290-H-0.1
[2,4,5,6-^2^H] Nicotinamide	Cambridge Isotope Laboratories	CAT# DLM-6883-0.5
Blotting grade blocker nonfat dry milk	Bio-Rad	CAT# 1706404XTU
RIPA lysis and extraction buffer	Thermo Fisher	CAT# 89900
Pierce BCA protein assay kits	Thermo Fisher	CAT# 23225
Xylene substitute	Carl Roth GmbH+ Co. KG	CAT# 6640.4
Hydrogen peroxide	Sigma Aldrich	CAT# H1009
Bovine Serum Albumin (BSA)	Carl Roth GmbH+ Co. KG	CAT# 1ETA
Hematoxylin	Sigma Aldrich	CAT# 51275
ROTI-Histokitt mounting medium	Carl Roth GmbH+ Co. KG	CAT# 6638.1

Critical commercial assays		

Mouse Lipocalin-2/NGAL	R&D Systems	CAT# DY185
cDNA synthesis kit	Quata bio	CAT# 101414-100
PerfeCTa qPCR FastMix	Quanta bio	CAT# 95078-012
DNeasy PowerSoil Kit	Qiagen	CAT# 47014
Vectastain ABC kit	Vector Laboratories	CAT# PK-4000
DAB peroxidase substrate kit	Vector Laboratories	CAT# PK-4100

Deposited data		

Metabolomics, isotope tracing, and metagenomics raw dataset	This paper	MSV000102197

Experimental models: Organisms/strains		

C57BL/6 (Catheter implanted in the right jugular vein)	Charles River Laboratories	N/A

Software and algorithms		

El-MAVEN software	Elucidata	https://www.elucidata.io/el-maven
AccuCor	GitHub	https://github.com/XiaoyangSu/AccuCor
Prism 10	GraphPad	https://www.graphpad.com/scientific-software/prism/
OmicsNet 2.0	Zhou et al.^74^	https://www.omicsnet.ca/OmicsNet/UploadView.xhtml
HUMAnN3	Beghini et al.^75^	v3.9
FastP	Chen et al.^76^	v0.24.0
MicrobeCensus	https://github.com/snayfach/MicrobeCensus	v1.1.1
Kneaddata	https://github.com/biobakery/kneaddata	v0.12.2
MetaPhlan	https://github.com/biobakery/metaphlan	v4.0.6
Vegan	https://github.com/vegandevs/vegan	v2.6-10
Ape	Paradis et al.^77^	v5.8-1

Other		

Laboratory rodent diet	LabDiet	CAT# 5053
Metagenomic DNA extraction	One Health Microbiome Center Co-Laboratory at Pennsylvania State University- GitHub	https://github.com/BisanzLab/OHMC_Colaboratory

## Highlights

- Colitis induces pathway-specific dysregulation of NAD^+^ metabolism
- Host tissues compensate for impaired *de novo* NAD^+^ synthesis via salvage pathways
- Colitis drives distinct NAD^+^ adaptations across tissues and gut microbiota

## References

[1] Shats I, Williams JG, Liu J, Makarov MV, Wu X, Lih FB, Deterding LJ, Lim C, Xu X, Randall TA, (2020). Bacteria Boost Mammalian Host NAD Metabolism by Engaging the Deamidated Biosynthesis Pathway. Cell Metab. 31, 564–579.e7. 10.1016/j.cmet.2020.02.001. PMC7194078 32130883

[2] Covarrubias AJ, Perrone R, Grozio A, and Verdin E (2021). NAD(+) metabolism and its roles in cellular processes during ageing. Nat. Rev. Mol. Cell Biol. 22, 119–141. 10.1038/s41580-020-00313-x. PMC7963035 33353981

[3] Belenky P, Bogan KL, and Brenner C (2007). NAD+ metabolism in health and disease. Trends Biochem. Sci. 32, 12–19. 10.1016/j.tibs.2006.11.006. 17161604

[4] McReynolds MR, Chellappa K, and Baur JA (2020). Age-related NAD(+) decline. Exp. Gerontol. 134, 110888. 10.1016/j.exger.2020.110888. PMC7442590 32097708

[5] Liu L, Su X, Quinn WJ 3rd, Hui S, Krukenberg K, Frederick DW, Redpath P, Zhan L, Chellappa K, White E, (2018). Quantitative Analysis of NAD Synthesis-Breakdown Fluxes. Cell Metab. 27, 1067–1080.e5. 10.1016/j.cmet.2018.03.018. PMC5932087 29685734

[6] Johnson S, and Imai SI (2018). NAD (+) biosynthesis, aging, and disease. F1000Res. 7, 132. 10.12688/f1000research.12120.1. PMC5795269 29744033

[7] Chellappa K, McReynolds MR, Lu W, Zeng X, Makarov M, Hayat F, Mukherjee S, Bhat YR, Lingala SR, Shima RT, (2022). NAD precursors cycle between host tissues and the gut microbiome. Cell Metab. 34, 1947–1959.e5. 10.1016/j.cmet.2022.11.004. PMC9825113 36476934

[8] Kang YH, Tucker SA, Quevedo SF, Inal A, Korzenik JR, and Haigis MC (2022). Metabolic analyses reveal dysregulated NAD+ metabolism and altered mitochondrial state in ulcerative colitis. PLoS One 17, e0273080. 10.1371/journal.pone.0273080. PMC9385040 35976971

[9] Novak EA, Crawford EC, Mentrup HL, Griffith BD, Fletcher DM, Flanagan MR, Schneider C, Firek B, Rogers MB, Morowitz MJ, (2023). Epithelial NAD(+) depletion drives mitochondrial dysfunction and contributes to intestinal inflammation. Front. Immunol. 14, 1231700. 10.3389/fimmu.2023.1231700. PMC10512956 37744380

[10] Taubenheim J, Kadibalban AS, Zimmermann J, Taubenheim C, Tran F, Schreiber S, Rosenstiel P, Aden K, and Kaleta C (2025). Author Correction: Metabolic modeling reveals a multi-level deregulation of host-microbiome metabolic networks in IBD. Nat. Commun. 16, 8978. 10.1038/s41467-025-64877-y. PMC12511306 41068162

[11] Nikolaus S, Schulte B, Al-Massad N, Thieme F, Schulte DM, Bethge J, Rehman A, Tran F, Aden K, Häsler R, (2017). Increased Tryptophan Metabolism Is Associated With Activity of Inflammatory Bowel Diseases. Gastroenterology 153, 1504–1516.e2. 10.1053/j.gastro.2017.08.028. 28827067

[12] Harris DMM, Szymczak S, Schuchardt S, Labrenz J, Tran F, Welz L, Graßhoff H, Zirpel H, Sümbül M, Oumari M, (2024). Tryptophan degradation as a systems phenomenon in inflammation - an analysis across 13 chronic inflammatory diseases. EBioMedicine 102, 105056. 10.1016/j.ebiom.2024.105056. PMC10943670 38471395

[13] Fakhoury M, Negrulj R, Mooranian A, and Al-Salami H (2014). Inflammatory bowel disease: clinical aspects and treatments. J. Inflamm. Res. 7, 113–120. 10.2147/JIR.S65979. PMC4106026 25075198

[14] Goethel A, Croitoru K, and Philpott DJ (2018). The interplay between microbes and the immune response in inflammatory bowel disease. J. Physiol. 596, 3869–3882. 10.1113/JP275396. PMC6117586 29806140

[15] Zheng D, Liwinski T, and Elinav E (2020). Interaction between microbiota and immunity in health and disease. Cell Res. 30, 492–506. 10.1038/s41422-020-0332-7. PMC7264227 32433595

[16] Lloyd-Price J, Arze C, Ananthakrishnan AN, Schirmer M, Avila-Pacheco J, Poon TW, Andrews E, Ajami NJ, Bonham KS, Brislawn CJ, (2019). Multi-omics of the gut microbial ecosystem in inflammatory bowel diseases. Nature 569, 655–662. 10.1038/s41586-019-1237-9. PMC6650278 31142855

[17] Bao LL, Yu YQ, González-Acera M, Patankar JV, Giessl A, Sturm G, Kühl AA, Atreya R, Erkert L, Gámez-Belmonte R, (2025). Epithelial OPA1 links mitochondrial fusion to inflammatory bowel disease. Sci. Transl. Med. 17, eadn8699. 10.1126/scitranslmed.adn8699. 39813315

[18] Shon WJ, Lee YK, Shin JH, Choi EY, and Shin DM (2015). Severity of DSS-induced colitis is reduced in Ido1-deficient mice with down-regulation of TLR-MyD88-NF-kB transcriptional networks. Sci. Rep. 5, 17305. 10.1038/srep17305. PMC4661522 26610689

[19] Hamabata T, Nakamura T, Masuko S, Maeda S, and Murata T (2018). Production of lipid mediators across different disease stages of dextran sodium sulfate-induced colitis in mice. J. Lipid Res. 59, 586–595. 10.1194/jlr.M079095. PMC5880491 29414763

[20] Taghipour N, Molaei M, Mosaffa N, Rostami-Nejad M, Asadzadeh Aghdaei H, Anissian A, Azimzadeh P, and Zali MR (2016). An experimental model of colitis induced by dextran sulfate sodium from acute progresses to chronicity in C57BL/6: correlation between conditions of mice and the environment. Gastroenterol. Hepatol. Bed Bench 9, 45–52. PMC4702041 26744614

[21] Spencer DM, Veldman GM, Banerjee S, Willis J, and Levine AD (2002). Distinct inflammatory mechanisms mediate early versus late colitis in mice. Gastroenterology 122, 94–105. 10.1053/gast.2002.30308. 11781285

[22] Kwon J, Lee C, Heo S, Kim B, and Hyun CK (2021). DSS-induced colitis is associated with adipose tissue dysfunction and disrupted hepatic lipid metabolism leading to hepatosteatosis and dyslipidemia in mice. Sci. Rep. 11, 5283. 10.1038/s41598-021-84761-1. PMC7935975 33674694

[23] Chassaing B, Srinivasan G, Delgado MA, Young AN, Gewirtz AT, and Vijay-Kumar M (2012). Fecal lipocalin 2, a sensitive and broadly dynamic non-invasive biomarker for intestinal inflammation. PLoS One 7, e44328. 10.1371/journal.pone.0044328. PMC3434182 22957064

[24] Yadav SK, Ito N, Mindur JE, Kumar H, Youssef M, Suresh S, Kulkarni R, Rosario Y, Balashov KE, Dhib-Jalbut S, and Ito K (2022). Fecal Lcn-2 level is a sensitive biological indicator for gut dysbiosis and intestinal inflammation in multiple sclerosis. Front. Immunol. 13, 1015372. 10.3389/fimmu.2022.1015372. PMC9634083 36341389

[25] Wehkamp L, Harris DMM, Kim NM, Alsaadi AI, Wu Q, Oumari M, Taubenheim J, Volk V, Credidio G, Koncina E, (2026). A metabolic constraint in de novo NAD+ synthesis drives mucosal inflammation in IBD. J. Crohns Colitis 20, jjag043. 10.1093/ecco-jcc/jjag043. PMC13223750 42223054

[26] Xue C, Li G, Zheng Q, Gu X, Shi Q, Su Y, Chu Q, Yuan X, Bao Z, Lu J, and Li L (2023). Tryptophan metabolism in health and disease. Cell Metab. 35, 1304–1326. 10.1016/j.cmet.2023.06.004. 37352864

[27] Miyamoto K, Sujino T, and Kanai T (2024). The tryptophan metabolic pathway of the microbiome and host cells in health and disease. Int. Immunol. 36, 601–616. 10.1093/intimm/dxae035. PMC11562643 38869080

[28] Yu F, Du Y, Li C, Zhang H, Lai W, Li S, Ye Z, Fu W, Li S, Li XG, and Luo D (2024). Association between metabolites in tryptophan-kynurenine pathway and inflammatory bowel disease: a two-sample Mendelian randomization. Sci. Rep. 14, 201. 10.1038/s41598-023-50990-9. PMC10761717 38167867

[29] Michaudel C, Danne C, Agus A, Magniez A, Aucouturier A, Spatz M, Lefevre A, Kirchgesner J, Rolhion N, Wang Y, (2023). Rewiring the altered tryptophan metabolism as a novel therapeutic strategy in inflammatory bowel diseases. Gut 72, 1296–1307. 10.1136/gutjnl-2022-327337. PMC10314090 36270778

[30] Badawy AAB, and Guillemin G (2019). The Plasma [Kynurenine]/[Tryptophan] Ratio and Indoleamine 2,3-Dioxygenase: Time for Appraisal. Int. J. Tryptophan Res. 12, 1178646919868978. 10.1177/1178646919868978. PMC6710706 31488951

[31] O’Connor JC, André C, Wang Y, Lawson MA, Szegedi SS, Lestage J, Castanon N, Kelley KW, and Dantzer R (2009). Interferon-gamma and tumor necrosis factor-alpha mediate the upregulation of indoleamine 2,3-dioxygenase and the induction of depressive-like behavior in mice in response to bacillus Calmette-Guerin. J. Neurosci. 29, 4200–4209. 10.1523/JNEUROSCI.5032-08.2009. PMC2835569 19339614

[32] Zelante T, Iannitti RG, Cunha C, De Luca A, Giovannini G, Pieraccini G, Zecchi R, D’Angelo C, Massi-Benedetti C, Fallarino F, (2013). Tryptophan catabolites from microbiota engage aryl hydrocarbon receptor and balance mucosal reactivity via interleukin-22. Immunity 39, 372–385. 10.1016/j.immuni.2013.08.003. 23973224

[33] Su X, Gao Y, and Yang R (2022). Gut Microbiota-Derived Tryptophan Metabolites Maintain Gut and Systemic Homeostasis. Cells 11, 2296. 10.3390/cells11152296. PMC9330295 35892593

[34] Li S (2023). Modulation of immunity by tryptophan microbial metabolites. Front. Nutr. 10, 1209613. 10.3389/fnut.2023.1209613. PMC10382180 37521424

[35] Frank DN, St Amand AL, Feldman RA, Boedeker EC, Harpaz N, and Pace NR (2007). Molecular-phylogenetic characterization of microbial community imbalances in human inflammatory bowel diseases. Proc. Natl. Acad. Sci. USA 104, 13780–13785. 10.1073/pnas.0706625104. PMC1959459 17699621

[36] Nagao-Kitamoto H, Shreiner AB, Gillilland MG 3rd, Kitamoto S, Ishii C, Hirayama A, Kuffa P, El-Zaatari M, Grasberger H, Seekatz AM, (2016). Functional Characterization of Inflammatory Bowel Disease-Associated Gut Dysbiosis in Gnotobiotic Mice. Cell. Mol. Gastroenterol. Hepatol. 2, 468–481. 10.1016/j.jcmgh.2016.02.003. PMC5042563 27795980

[37] Berry D, Kuzyk O, Rauch I, Heider S, Schwab C, Hainzl E, Decker T, Müller M, Strobl B, Schleper C, (2015). Intestinal Microbiota Signatures Associated with Inflammation History in Mice Experiencing Recurring Colitis. Front. Microbiol. 6, 1408. 10.3389/fmicb.2015.01408. PMC4678223 26697002

[38] Mandić AD, Woting A, Jaenicke T, Sander A, Sabrowski W, Rolle-Kampcyk U, von Bergen M, and Blaut M (2019). Clostridium ramosum regulates enterochromaffin cell development and serotonin release. Sci. Rep. 9, 1177. 10.1038/s41598-018-38018-z. PMC6362283 30718836

[39] Wang Y, Li J, Yang Q, Zhu Z, Cheng F, Ai X, Liu Y, Zhao D, Zhao F, and Cheng P (2024). 5-Methoxytryptophan Alleviates Dextran Sulfate Sodium-Induced Colitis by Inhibiting the Intestinal Epithelial Damage and Inflammatory Response. Mediat. Inflamm. 2024, 1484806. 10.1155/2024/1484806. PMC11390199 39262415

[40] Wang YF, Hsu YJ, Wu HF, Lee GL, Yang YS, Wu JY, Yet SF, Wu KK, and Kuo CC (2016). Endothelium-Derived 5-Methoxytryptophan Is a Circulating Anti-Inflammatory Molecule That Blocks Systemic Inflammation. Circ. Res. 119, 222–236. 10.1161/CIRCRESAHA.116.308559. 27151398

[41] Maassen S, Warner H, Ioannidis M, Jansma J, Markus H, El Aidy S, Chiara MD, Chiara JL, Maierhofer L, Weavers H, and van den Bogaart G (2022). Mitochondrial interaction of fibrosis-protective 5-methoxy tryptophan enhances collagen uptake by macrophages. Free Radic. Biol. Med. 188, 287–297. 10.1016/j.freerad-biomed.2022.06.235. PMC7614693 35753585

[42] Scott SA, Fu J, and Chang PV (2020). Microbial tryptophan metabolites regulate gut barrier function via the aryl hydrocarbon receptor. Proc. Natl. Acad. Sci. USA 117, 19376–19387. 10.1073/pnas.2000047117. PMC7431026 32719140

[43] Alexeev EE, Lanis JM, Kao DJ, Campbell EL, Kelly CJ, Battista KD, Gerich ME, Jenkins BR, Walk ST, Kominsky DJ, and Colgan SP (2018). Microbiota-Derived Indole Metabolites Promote Human and Murine Intestinal Homeostasis through Regulation of Interleukin-10 Receptor. Am. J. Pathol. 188, 1183–1194. 10.1016/j.ajpath.2018.01.011. PMC5906738 29454749

[44] Lee JH, and Lee J (2010). Indole as an intercellular signal in microbial communities. FEMS Microbiol. Rev. 34, 426–444. 10.1111/j.1574-6976.2009.00204.x. 20070374

[45] Tennoune N, Andriamihaja M, and Blachier F (2022). Production of Indole and Indole-Related Compounds by the Intestinal Microbiota and Consequences for the Host: The Good, the Bad, and the Ugly. Microorganisms 10, 930. 10.3390/microorganisms10050930. PMC9145683 35630374

[46] Shimada Y, Kinoshita M, Harada K, Mizutani M, Masahata K, Kayama H, and Takeda K (2013). Commensal bacteria-dependent indole production enhances epithelial barrier function in the colon. PLoS One 8, e80604. 10.1371/journal.pone.0080604. PMC3835565 24278294

[47] Bhattarai Y, Jie S, Linden DR, Ghatak S, Mars RAT, Williams BB, Pu M, Sonnenburg JL, Fischbach MA, Farrugia G, (2020). Bacterially Derived Tryptamine Increases Mucus Release by Activating a Host Receptor in a Mouse Model of Inflammatory Bowel Disease. iScience 23, 101798. 10.1016/j.isci.2020.101798. PMC7702010 33299969

[48] Bhattarai Y, Williams BB, Battaglioli EJ, Whitaker WR, Till L, Grover M, Linden DR, Akiba Y, Kandimalla KK, Zachos NC, (2018). Gut Microbiota-Produced Tryptamine Activates an Epithelial G-Protein-Coupled Receptor to Increase Colonic Secretion. Cell Host Microbe 23, 775–785.e5. 10.1016/j.chom.2018.05.004. PMC6055526 29902441

[49] Zhai L, Xiao H, Lin C, Wong HLX, Lam YY, Gong M, Wu G, Ning Z, Huang C, Zhang Y, (2023). Gut microbiota-derived tryptamine and phenethylamine impair insulin sensitivity in metabolic syndrome and irritable bowel syndrome. Nat. Commun. 14, 4986. 10.1038/s41467-023-40552-y. PMC10435514 37591886

[50] Adriouch S, Hubert S, Pechberty S, Koch-Nolte F, Haag F, and Seman M (2007). NAD+ released during inflammation participates in T cell homeostasis by inducing ART2-mediated death of naive T cells in vivo. J. Immunol. 179, 186–194. 10.4049/jimmunol.179.1.186. 17579037

[51] Han X, Uchiyama T, Sappington PL, Yaguchi A, Yang R, Fink MP, and Delude RL (2003). NAD+ ameliorates inflammation-induced epithelial barrier dysfunction in cultured enterocytes and mouse ileal mucosa. J. Pharmacol. Exp. Therapeut. 307, 443–449. 10.1124/jpet.103.056556. 12975482

[52] Taubenheim J, Kadibalban AS, Zimmermann J, Taubenheim C, Tran F, Schreiber S, Rosenstiel P, Aden K, and Kaleta C (2025). Metabolic modeling reveals a multi-level deregulation of host-microbiome metabolic networks in IBD. Nat. Commun. 16, 5120. 10.1038/s41467-025-60233-2. PMC12130198 40456745

[53] Schreiber S, Waetzig GH, López-Agudelo VA, Geisler C, Schlicht K, Franzenburg S, di Giuseppe R, Pape D, Bahmer T, Krawczak M, (2025). Nicotinamide modulates gut microbial metabolic potential and accelerates recovery in mild-to-moderate COVID-19. Nat. Metab. 7, 1136–1149. 10.1038/s42255-025-01290-1. PMC12198012 40355744

[54] McReynolds MR, Chellappa K, Chiles E, Jankowski C, Shen Y, Chen L, Descamps HC, Mukherjee S, Bhat YR, Lingala SR, (2021). NAD(+) flux is maintained in aged mice despite lower tissue concentrations. Cell Syst. 12, 1160–1172.e4. 10.1016/j.cels.2021.09.001. PMC8678178 34559996

[55] Real AM, Hong S, and Pissios P (2013). Nicotinamide N-oxidation by CYP2E1 in human liver microsomes. Drug Metab. Dispos. 41, 550–553. 10.1124/dmd.112.049734. PMC3583486 23418369

[56] Hwang ES, and Song SB (2017). Nicotinamide is an inhibitor of SIRT1 in vitro, but can be a stimulator in cells. Cell. Mol. Life Sci. 74, 3347–3362. 10.1007/s00018-017-2527-8. PMC11107671 28417163

[57] Lauritzen KH, Olsen MB, Ahmed MS, Yang K, Rinholm JE, Bergersen LH, Esbensen QY, Sverkeli LJ, Ziegler M, Attramadal H, (2021). Instability in NAD(+) metabolism leads to impaired cardiac mitochondrial function and communication. eLife 10, e59828. 10.7554/eLife.59828. PMC8331182 34343089

[58] Niño-Narvión J, Rojo-López MI, Martinez-Santos P, Rossell J, Ruiz-Alcaraz AJ, Alonso N, Ramos-Molina B, Mauricio D, and Julve J (2023). NAD+ Precursors and Intestinal Inflammation: Therapeutic Insights Involving Gut Microbiota. Nutrients 15, 2992. 10.3390/nu15132992. PMC10346866 37447318

[59] Wnorowski A, Wnorowska S, Kurzepa J, and Parada-Turska J (2021). Alterations in Kynurenine and NAD(+) Salvage Pathways during the Successful Treatment of Inflammatory Bowel Disease Suggest HCAR3 and NNMT as Potential Drug Targets. Int. J. Mol. Sci. 22, 13497. 10.3390/ijms222413497. PMC8705244 34948292

[60] Chini CCS, Cordeiro HS, Tran NLK, and Chini EN (2024). NAD metabolism: Role in senescence regulation and aging. Aging Cell 23, e13920. 10.1111/acel.13920. PMC10776128 37424179

[61] Amjad S, Nisar S, Bhat AA, Shah AR, Frenneaux MP, Fakhro K, Haris M, Reddy R, Patay Z, Baur J, and Bagga P (2021). Role of NAD(+) in regulating cellular and metabolic signaling pathways. Mol. Metabol. 49, 101195. 10.1016/j.molmet.2021.101195. PMC7973386 33609766

[62] Navas LE, and Carnero A (2021). NAD(+) metabolism, stemness, the immune response, and cancer. Signal Transduct. Targeted Ther. 6, 2. 10.1038/s41392-020-00354-w. PMC7775471 33384409

[63] Cantó C, Menzies KJ, and Auwerx J (2015). NAD(+) Metabolism and the Control of Energy Homeostasis: A Balancing Act between Mitochondria and the Nucleus. Cell Metab. 22, 31–53. 10.1016/j.cmet.2015.05.023. PMC4487780 26118927

[64] Badawy AAB (2017). Kynurenine Pathway of Tryptophan Metabolism: Regulatory and Functional Aspects. Int. J. Tryptophan Res. 10, 1178646917691938. 10.1177/1178646917691938. PMC5398323 28469468

[65] Proietti E, Pauwels RWM, de Vries AC, Orecchini E, Volpi C, Orabona C, Peppelenbosch MP, Fuhler GM, and Mondanelli G (2023). Modulation of Indoleamine 2,3-Dioxygenase 1 During Inflammatory Bowel Disease Activity in Humans and Mice. Int. J. Tryptophan Res. 16, 11786469231153109. 10.1177/11786469231153109. PMC9926376 36798536

[66] Salazar F, Awuah D, Negm OH, Shakib F, and Ghaemmaghami AM (2017). The role of indoleamine 2,3-dioxygenase-aryl hydrocarbon receptor pathway in the TLR4-induced tolerogenic phenotype in human DCs. Sci. Rep. 7, 43337. 10.1038/srep43337. PMC5335671 28256612

[67] Neurath MF (2024). Strategies for targeting cytokines in inflammatory bowel disease. Nat. Rev. Immunol. 24, 559–576. 10.1038/s41577-024-01008-6. 38486124

[68] Hu T, Liu CH, Lei M, Zeng Q, Li L, Tang H, and Zhang N (2024). Metabolic regulation of the immune system in health and diseases: mechanisms and interventions. Signal Transduct. Targeted Ther. 9, 268. 10.1038/s41392-024-01954-6. PMC11461632 39379377

[69] Guggeis MA, Harris DM, Welz L, Rosenstiel P, and Aden K (2025). Microbiota-derived metabolites in inflammatory bowel disease. Semin. Immunopathol. 47, 19. 10.1007/s00281-025-01046-9. PMC11876236 40032666

[70] Hu S, Bourgonje AR, Gacesa R, Jansen BH, Björk JR, Bangma A, Hidding IJ, van Dullemen HM, Visschedijk MC, Faber KN, (2024). Mucosal host-microbe interactions associate with clinical phenotypes in inflammatory bowel disease. Nat. Commun. 15, 1470. 10.1038/s41467-024-45855-2. PMC10874382 38368394

[71] Magnúsdóttir S, Ravcheev D, de Crécy-Lagard V, and Thiele I (2015). Systematic genome assessment of B-vitamin biosynthesis suggests co-operation among gut microbes. Front. Genet. 6, 148. 10.3389/fgene.2015.00148. PMC4403557 25941533

[72] Gazzaniga F, Stebbins R, Chang SZ, McPeek MA, and Brenner C (2009). Microbial NAD metabolism: lessons from comparative genomics. Microbiol. Mol. Biol. Rev. 73, 529–541. 10.1128/MMBR.00042-08. PMC2738131 19721089

[73] Zhang H, Ryu D, Wu Y, Gariani K, Wang X, Luan P, D’Amico D, Ropelle ER, Lutolf MP, Aebersold R, (2016). NAD(+) repletion improves mitochondrial and stem cell function and enhances life span in mice. Science 352, 1436–1443. 10.1126/science.aaf2693. 27127236

[74] Zhou G, Pang Z, Lu Y, Ewald J, and Xia J (2022). OmicsNet 2.0: a web-based platform for multi-omics integration and network visual analytics. Nucleic Acids Res. 50, W527–W533. 10.1093/nar/gkac376. PMC9252810 35639733

[75] Beghini F, McIver LJ, Blanco-Míguez A, Dubois L, Asnicar F, Maharjan S, Mailyan A, Manghi P, Scholz M, Thomas AM, (2021). Integrating taxonomic, functional, and strain-level profiling of diverse microbial communities with bioBakery 3. eLife 10, e65088. 10.7554/eLife.65088. PMC8096432 33944776

[76] Chen S, Zhou Y, Chen Y, and Gu J (2018). fastp: an ultra-fast all-in-one FASTQ preprocessor. Bioinformatics 34, i884–i890. 10.1093/bioinformatics/bty560. PMC6129281 30423086

[77] Paradis E, Claude J, and Strimmer K (2004). APE: Analyses of Phylogenetics and Evolution in R language. Bioinformatics 20, 289–290. 10.1093/bioinformatics/btg412. 14734327

[78] Rangan P, Choi I, Wei M, Navarrete G, Guen E, Brandhorst S, Enyati N, Pasia G, Maesincee D, Ocon V, (2019). Fasting-Mimicking Diet Modulates Microbiota and Promotes Intestinal Regeneration to Reduce Inflammatory Bowel Disease Pathology. Cell Rep. 26, 2704–2719.e6. 10.1016/j.celrep.2019.02.019. PMC6528490 30840892

[79] Erben U, Loddenkemper C, Doerfel K, Spieckermann S, Haller D, Heimesaat MM, Zeitz M, Siegmund B, and Kühl AA (2014). A guide to histomorphological evaluation of intestinal inflammation in mouse models. Int. J. Clin. Exp. Pathol. 7, 4557–4576. PMC4152019 25197329

[80] Chen L, Lu W, Wang L, Xing X, Chen Z, Teng X, Zeng X, Muscarella AD, Shen Y, Cowan A, (2021). Metabolite discovery through global annotation of untargeted metabolomics data. Nat. Methods 18, 1377–1385. 10.1038/s41592-021-01303-3. PMC8733904 34711973

[81] Lu W, Xing X, Wang L, Chen L, Zhang S, McReynolds MR, and Rabinowitz JD (2020). Improved Annotation of Untargeted Metabolomics Data through Buffer Modifications That Shift Adduct Mass and Intensity. Anal. Chem. 92, 11573–11581. 10.1021/acs.analchem.0c00985. PMC7484094 32614575

[82] Wang L, Xing X, Chen L, Yang L, Su X, Rabitz H, Lu W, and Rabinowitz JD (2019). Peak Annotation and Verification Engine for Untargeted LC-MS Metabolomics. Anal. Chem. 91, 1838–1846. 10.1021/acs.analchem.8b03132. PMC6501219 30586294

[83] Su X, Lu W, and Rabinowitz JD (2017). Metabolite Spectral Accuracy on Orbitraps. Anal. Chem. 89, 5940–5948. 10.1021/acs.analchem.7b00396. PMC5748891 28471646

[84] McIver LJ, Abu-Ali G, Franzosa EA, Schwager R, Morgan XC, Waldron L, Segata N, and Huttenhower C (2018). bioBakery: a meta’omic analysis environment. Bioinformatics 34, 1235–1237. 10.1093/bioinformatics/btx754. PMC6030947 29194469

[85] Nayfach S, and Pollard KS (2015). Average genome size estimation improves comparative metagenomics and sheds light on the functional ecology of the human microbiome. Genome Biol. 16, 51. 10.1186/s13059-015-0611-7. PMC4389708 25853934

