# Supplementary material for: Tracing NAD+ metabolism uncovers adaptive coordination between host and microbiome during colitis
Source: Cell Rep. Author manuscript; Available in PMC 2026 Sep 28. (PMC13619250; doi:10.1016/j.celrep.2026.117730)
Supplement: Supplementary file 1 [file NIHMS2206913-supplement-1.pdf]

Cell Reports, Volume *45*

Supplemental information

Tracing NAD^+ metabolism uncovers adaptive coordination between host and microbiome during colitis

Abrar I. Alsaadi, Lina Wehkamp, Anant A. Pothakamury, Mahmoud S. Yahia, Praveena Prasad, Clara Gilloteau, Jaedon Sadler, Jaclyn D. Smith, Brenita C. Jenkins, Garret S. Diven, Johanna Bornhäuser, Taous Mekdoud, Shuchang Tian, Philip Rosenstiel, Stefan Schreiber, Jordan E. Bisanz, Konrad Aden, and Melanie R. McReynolds

Fig.S1

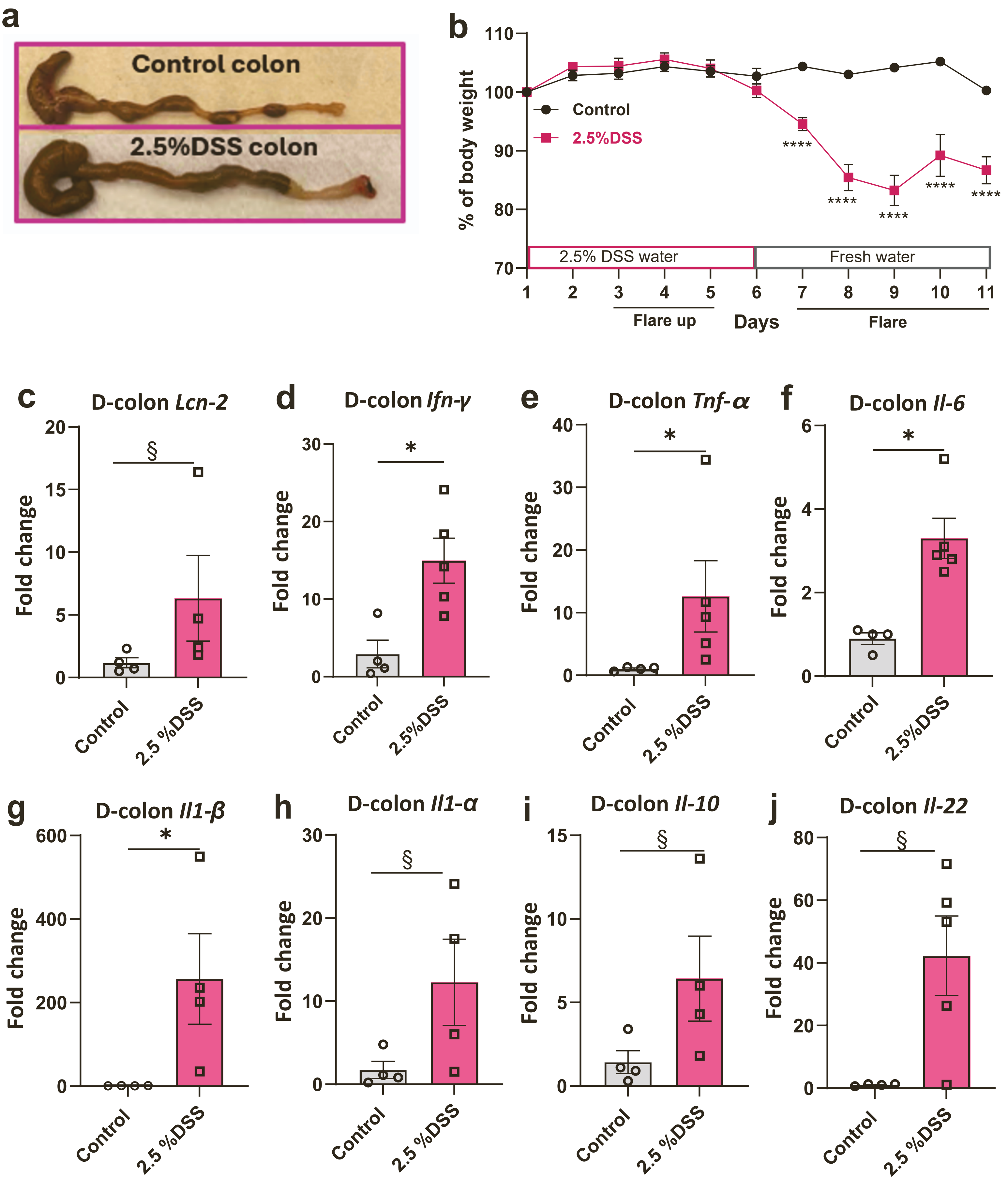
a
Control colon
2.5%DSS colon
b
% of body weight
110
100
90
80
70
Control
2.5%DSS
****
2.5% DSS water
Fresh water
1
2
3
4
5
6
7
8
9
10
11
Flare up
Days
Flare
c
D-colon Lcn-2
Fold change
§
Control
2.5 %DSS
d
D-colon Ifn-γ
*
2.5%DSS
e
D-colon Tnf-α
f
D-colon Il-6
g
D-colon Il1-β
h
D-colon Il1-α
i
D-colon Il-10
j
D-colon Il-22

Fig.S1| Assessment of colitis induction in mice.

(a) Representative image of a non-inflamed colon from control (day 8; upper panel) compared to an inflamed colon from mice treated with 2.5% DSS dissolved in drinking water (day 8; lower panel). (**b**) Percent change in body weight compared to initial body weight during experimental colitis induction. Inflammatory markers mRNA expression was measured by qRT-PCR in the distal colon tissues from DSS-treated mice during the active flare phase (days 8 and 11) and control mice, normalized to (*Tbp*); **(c)** Lipocalin-2 (*Lcn-2*), (**d**) Interferon gamma *(Ifn-γ)* (**e**) *Tumor necrosis factor alpha (Tnfα)*, (**f**) Interleukin 6 *(Il-6)*, (**g**) Interleukin-1 beta *(Il-1β*), (**h**) Interleukin-1 alpha (*Il-1α*), and anti-inflammatory cytokines, (**i**) Interleukin-10 (*Il-10*) and (**j**) Interleukin-22 (*Il-22*). Data are presented as mean ± SEM, in (**b**) (n=4-12) and (**c-j**) (n=4-5). Mann-Whitney U test for two-groups comparisons. § <0.1, *P<0.05, **P<0.01,***P<0.001, and ****P<0.0001.

Fig.S2

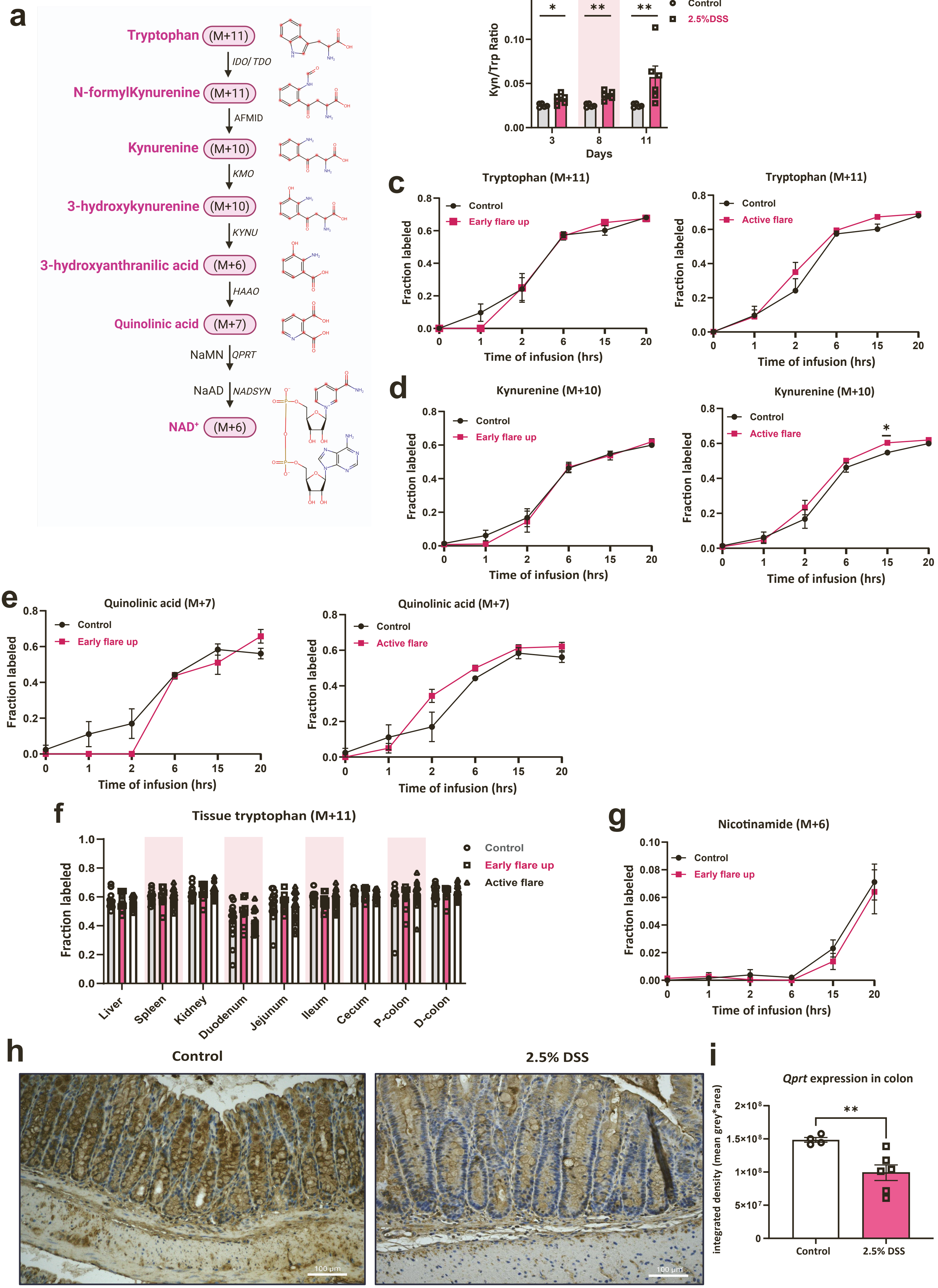
a
Tryptophan (M+11)
IDO/ TDO
N-formylKynurenine (M+11)
AFMID
Kynurenine (M+10)
KMO
3-hydroxykynurenine (M+10)
KYNU
3-hydroxyanthranilic acid (M+6)
HAAO
Quinolinic acid (M+7)
NaMN QPRT
NaAD NADSYN
NAD+ (M+6)
b
Circulation
Flare up
Flare
Control
2.5%DSS
Kyn/Trp Ratio
Days
c
Tryptophan (M+11)
Control
Early flare up
Active flare
Fraction labeled
Time of infusion (hrs)
d
Kynurenine (M+10)
Control
Early flare up
Active flare
Fraction labeled
Time of infusion (hrs)
e
Quinolinic acid (M+7)
Control
Early flare up
Active flare
Fraction labeled
Time of infusion (hrs)
f
Tissue tryptophan (M+11)
Control
Early flare up
Active flare
Fraction labeled
Liver
Spleen
Kidney
Duodenum
Jejunum
Ileum
Cecum
P-colon
D-colon
g
Nicotinamide (M+6)
Control
Early flare up
Fraction labeled
Time of infusion (hrs)
h
Control
2.5% DSS
100 μm
i
Qprt expression in colon
integrated density (mean grey*area)
Control
2.5% DSS

Fig.S2| Metabolic analysis of tryptophan and its downstream metabolites in the serum and tissues during acute intestinal inflammation.

(a) Schematic of tryptophan metabolism through the kynurenine pathway (M+n) M represents the mass of the labeled metabolite and the n is the number of labeled carbons. (**b**) LC-MS measurement of the total abundance of the kynurenine-to-tryptophan ratio (Kyn/Trp) in the circulation of mice treated with 2.5% DSS compared to control mice (n=4-5). (**c-e**) LC-MS analysis of labeled fractions of tryptophan, kynurenine, and quinolinic acid in the serum during the early flare up (left) and active flare (right) phases. (**f**) Tissue fraction labeled of tryptophan. (**g**) Serum fractional labeling of nicotinamide over 20-hour intravenous infusion during the early flare up phase of intestinal inflammation. (**h**) Immunohistochemical (IHC) staining of *Qprt* in Swiss roll sections of the distal colon from 2.5% DSS-treated mice and control (at magnification 40x, scale bar 100µm). (**i**) Quantification of *qprt* expression in 2.5% DSS-treated and control groups. Data are presented as mean ± SEM; in (**a**) (n=6 in each group), (**c-g**) (n=10-20). Statistical significance was determined by Mann-Whitney U test used for comparisons between two groups, and the Kruskal-Wallis test followed by Dunn's post hoc test for comparisons among more than two groups. *P<0.05 and **P<0.01.

Fig.S3

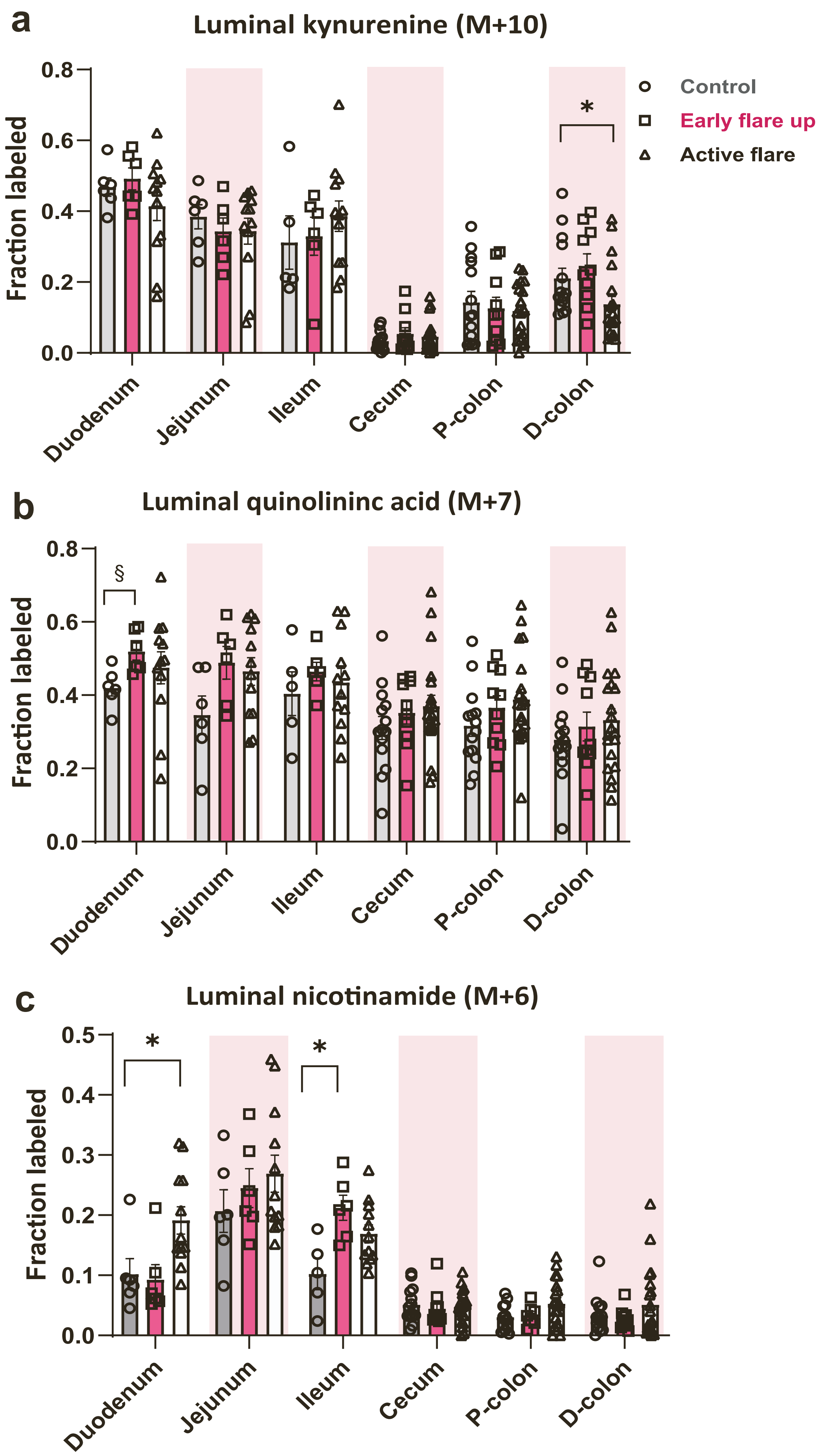
a
Luminal kynurenine (M+10)
Fraction labeled
0.8
0.6
0.4
0.2
0.0
*
Control
Early flare up
Active flare
Duodenum
Jejunum
Ileum
Cecum
P-colon
D-colon
b
Luminal quinolininc acid (M+7)
Fraction labeled
0.8
0.6
0.4
0.2
0.0
§
Duodenum
Jejunum
Ileum
Cecum
P-colon
D-colon
c
Luminal nicotinamide (M+6)
Fraction labeled
0.5
0.4
0.3
0.2
0.1
0.0
*
*
Duodenum
Jejunum
Ileum
Cecum
P-colon
D-colon

Fig.S3| Gut luminal tryptophan downstream metabolites via the kynurenine pathway.

The fractional labeled of luminal metabolites derived from tryptophan tracer measured by LC-MS of (**a**) kynurenine, (**b**) quinolinic acid, (**c**) nicotinamide in DSS-treated mice compared to control. Data are presented as mean ± SEM (n=10-20). Statistical significance was determined by Kruskal-Wallis test followed by Dunn's post hoc test for comparisons among more than two groups. § <0.1 and *P<0.05.

Fig.S4

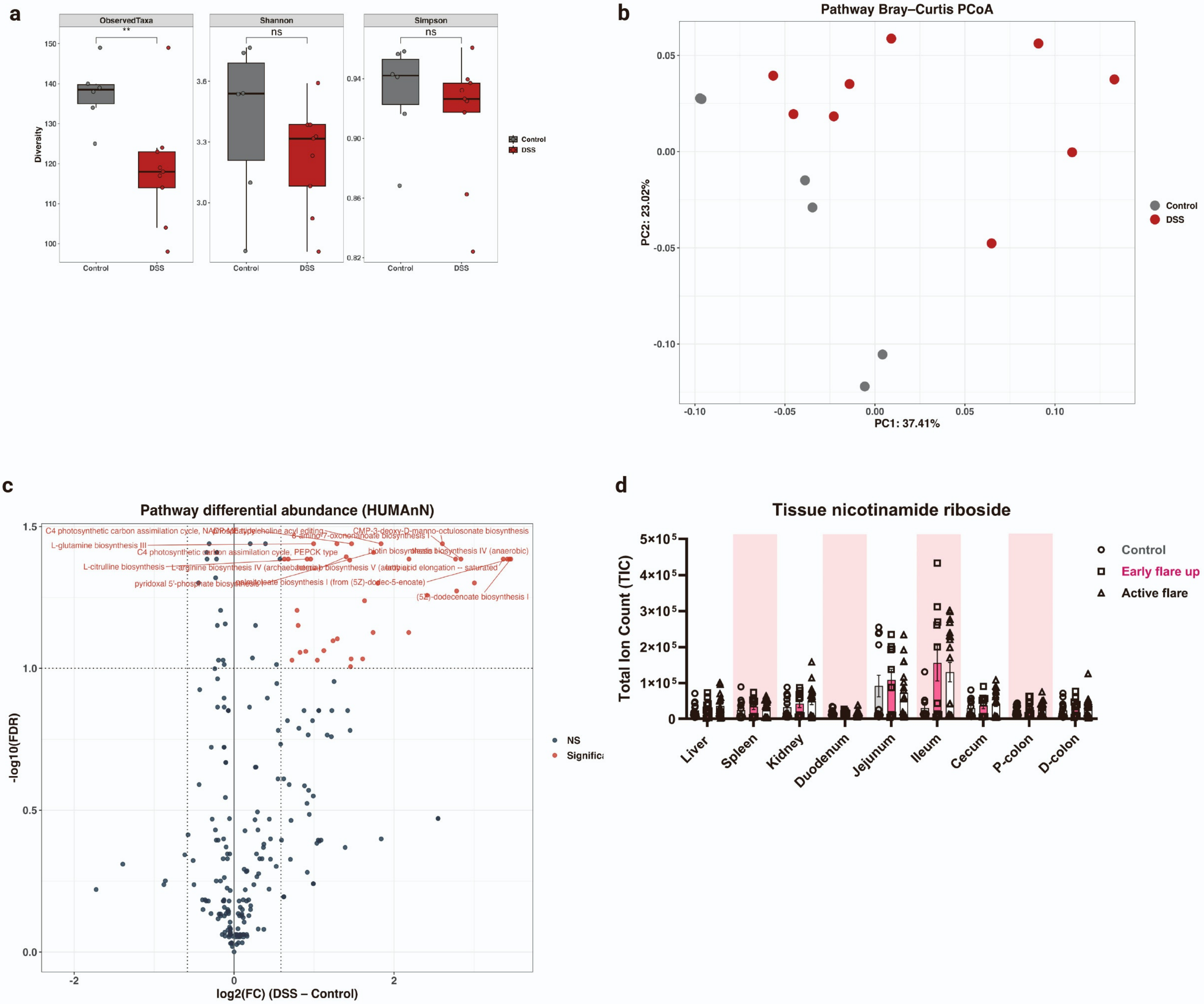

a
ObservedTaxa
Shannon
Simpson
**
ns
ns
Diversity
Control
DSS
b
Pathway Bray–Curtis PCoA
PC1: 37.41%
PC2: 23.02%
Control
DSS
c
Pathway differential abundance (HUMAnN)
log2(FC) (DSS – Control)
-log10(FDR)
NS
Significa
C4 photosynthetic carbon assimilation cycle, NADP-ME type
L-glutamine biosynthesis III
C4 photosynthetic carbon assimilation cycle, PEPCK type
L-citrulline biosynthesis
L-arginine biosynthesis IV (archaebacteria)
pyridoxal 5'-phosphate biosynthesis I
CMP-3-deoxy-D-manno-octulosonate biosynthesis
8-amino-7-oxononanoate biosynthesis I
biotin biosynthesis I
fatty acid elongation -- saturated
palmitoleate biosynthesis I (from (5Z)-dodec-5-enoate)
(5Z)-dodecenoate biosynthesis I
d
Tissue nicotinamide riboside
Total Ion Count (TIC)
Control
Early flare up
Active flare
Liver
Spleen
Kidney
Duodenum
Jejunum
Ileum
Cecum
P-colon
D-colon

Fig.S4| Pathway-level functional diversity and differential abundance analysis of DSS-induced colitis microbiomes.

(a) Alpha-diversity indices calculated using species-level taxonomic abundances (Observed Species Richness, Shannon's, Simpson's) show a significant reduction in observed richness in DSS-treated mice during the active phase (days 8 and 11) ($p = 0.0097$, Welch's t-test) but no significant change observed in Shannon or Simpson diversity, indicating loss of low-abundance species without major shifts in evenness. **(b)** Principal coordinate analysis (PCoA) of Bray-Curtis dissimilarities reveal clear separation between control (gray) and DSS (red) groups (PERMANOVA, $R^2 = 0.21$, $p = 0.007$), suggesting significant restructuring of pathway-level functional potential. **(c)** Volcano plot of differential pathway abundance (HUMAnN) comparing DSS versus control groups. Of all pathways analyzed, significantly altered features (FDR < 0.1, $|\log_2 FC| > 0.5$) are highlighted in red. **(d)** LC-MS relative total ion count (TIC) levels of tissue nicotinamide riboside in DSS-treated mice compared to control. Data are presented as mean ± SEM in **(d)** (n=10-20). Statistical significance was determined by Kruskal-Wallis test with post hoc Dunn's test for comparisons among more than two groups. NS, not significant, § <0.1, *P<0.05, **P<0.01, ***P<0.001, and ****P<0.0001.

Fig.S5

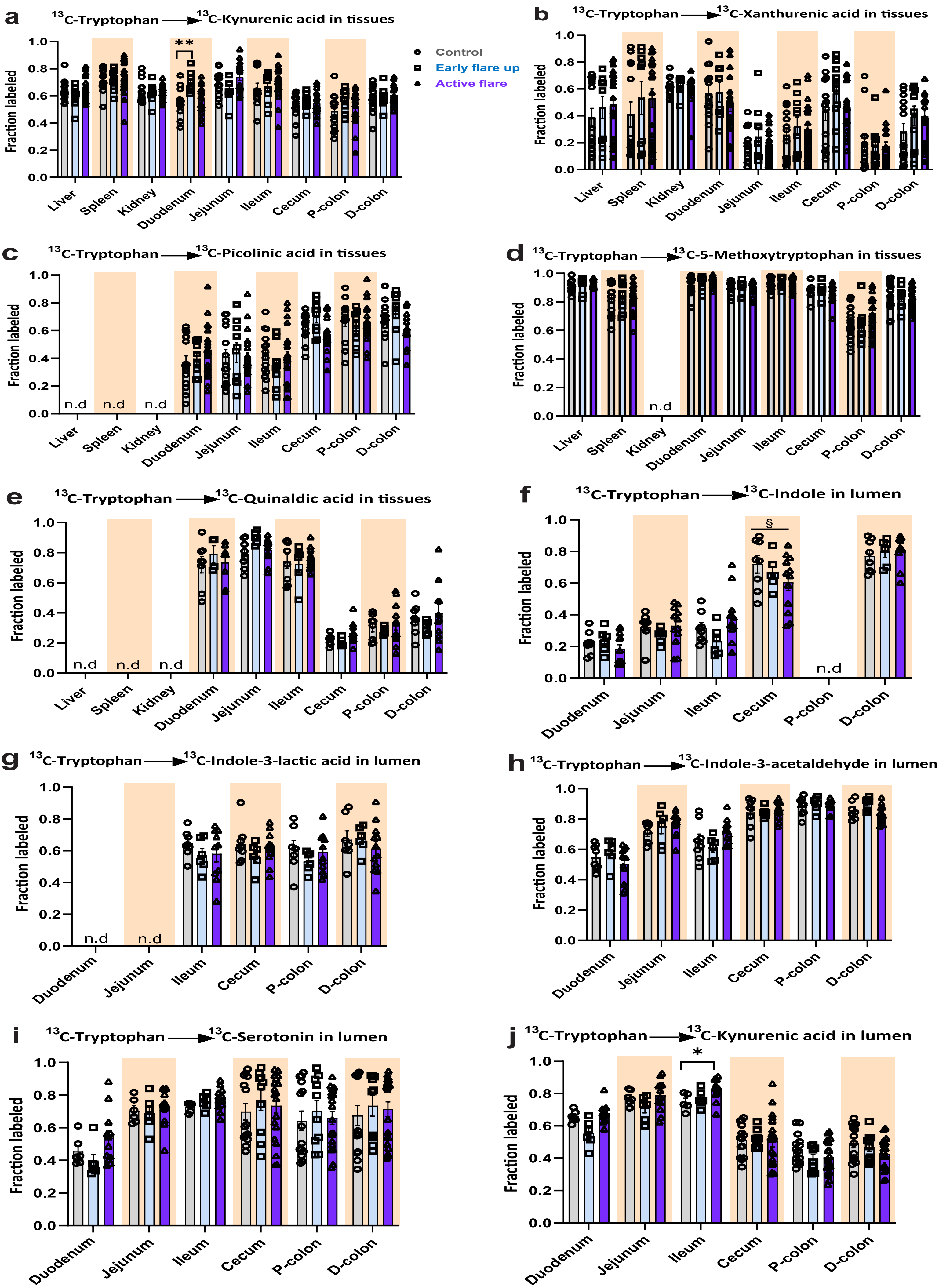

a
^{13}C-Tryptophan → ^{13}C-Kynurenic acid in tissues
Control
Early flare up
Active flare
Fraction labeled
Liver
Spleen
Kidney
Duodenum
Jejunum
Ileum
Cecum
P-colon
D-colon
b
^{13}C-Tryptophan → ^{13}C-Xanthurenic acid in tissues
c
^{13}C-Tryptophan → ^{13}C-Picolinic acid in tissues
n.d
d
^{13}C-Tryptophan → ^{13}C-5-Methoxytryptophan in tissues
e
^{13}C-Tryptophan → ^{13}C-Quinaldic acid in tissues
f
^{13}C-Tryptophan → ^{13}C-Indole in lumen
g
^{13}C-Tryptophan → ^{13}C-Indole-3-lactic acid in lumen
h
^{13}C-Tryptophan → ^{13}C-Indole-3-acetaldehyde in lumen
i
^{13}C-Tryptophan → ^{13}C-Serotonin in lumen
j
^{13}C-Tryptophan → ^{13}C-Kynurenic acid in lumen

Fig.S5| Quantification of tryptophan-derived pathways in host tissues and gut lumen.

Tracing the incorporation of ($^{13}C_{11}$-tryptophan) into different tryptophan-derived metabolites following 20-hour intravenous infusion, measured by LC-MS in the host tissues, (**a**) kynurenic acid, (**b**) xanthurenic acid, (**c**) picolinic acid, (**d**) 5-methoxytryptophan, (**e**) quinaldic acid; and in the gut lumen, (**f**) indole, (**g**) indole-3-lactic acid, (**h**) indole-3-acetaldehyde, (**i**) serotonin, (**j**) kynurenic acid. Data are presented as mean ± SEM (n=10-20). Statistical significance was determined by Kruskal-Wallis test followed by Dunn's post hoc test for comparisons among more than two groups. § <0.1, *P<0.05, **P<0.01, ***P<0.001, and ****P<0.0001. nd= not detected.

Fig.S6

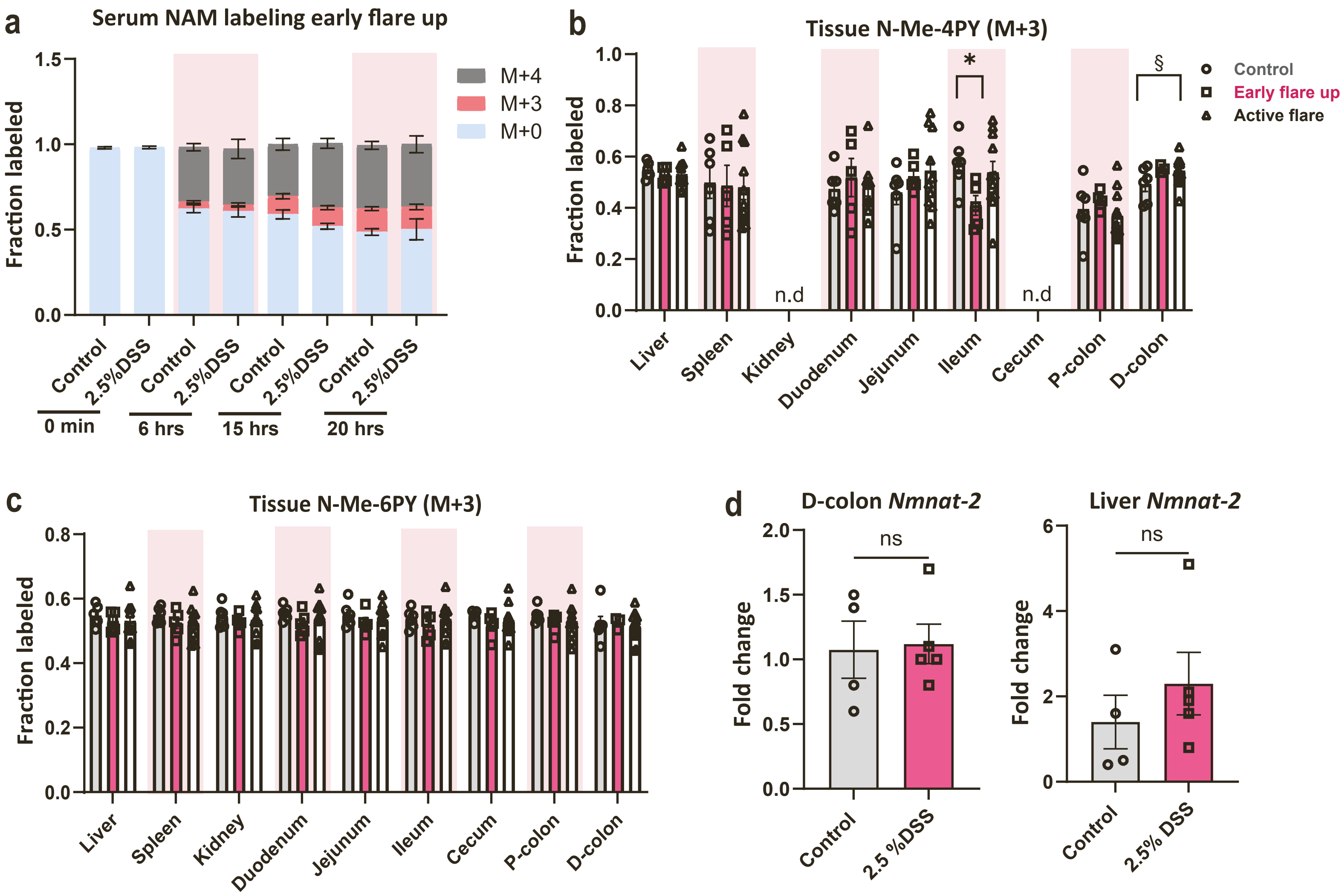

a
Serum NAM labeling early flare up
Fraction labeled
1.5
1.0
0.5
0.0
M+4
M+3
M+0
Control
2.5%DSS
Control
2.5%DSS
Control
2.5%DSS
Control
2.5%DSS
0 min
6 hrs
15 hrs
20 hrs
b
Tissue N-Me-4PY (M+3)
Fraction labeled
1.0
0.8
0.6
0.4
0.2
0.0
*
§
n.d
n.d
Control
Early flare up
Active flare
Liver
Spleen
Kidney
Duodenum
Jejunum
Ileum
Cecum
P-colon
D-colon
c
Tissue N-Me-6PY (M+3)
Fraction labeled
0.8
0.6
0.4
0.2
0.0
Liver
Spleen
Kidney
Duodenum
Jejunum
Ileum
Cecum
P-colon
D-colon
d
D-colon Nmnat-2
Fold change
2.0
1.5
1.0
0.5
0.0
ns
Control
2.5 %DSS
Liver Nmnat-2
Fold change
6
4
2
0
ns
Control
2.5% DSS

Fig.S6| Metabolic profiling of metabolites produced via the salvage pathway from nicotinamide.

(a) Fractional labeling of nicotinamide in the circulation over 20-hour intravenous infusion of (2,4,5,6-$^{2}H_4$-NAM) during the early flare up phase (M+0: unlabeled NAM, M+3: recycled NAM, M+4: infusate NAM). Fraction labeled of the methylnicotinamide byproducts in host tissues, (**b**) N-Me-4PY and (**c**) N-Me-6PY. (**d**) qRT-PCR analysis of mRNA expression normalized to *Tbp* in distal colon and liver tissues during the active flare phase (days 8 and 11) compared to control of nicotinamide mononucleotide adenylyltransferase 2 (*NMNAT 2*; cytoplasmic). Data are presented as mean ± SEM, in **a-c** (n=8-18) and in **d** (n=45). Statistical significance was determined by Mann-Whitney U test used for comparisons between two groups, and the Kruskal-Wallis test followed by Dunn's post hoc test for comparisons among more than two groups. § <0.1, *P<0.05, ns= not significant.

Fig.S7

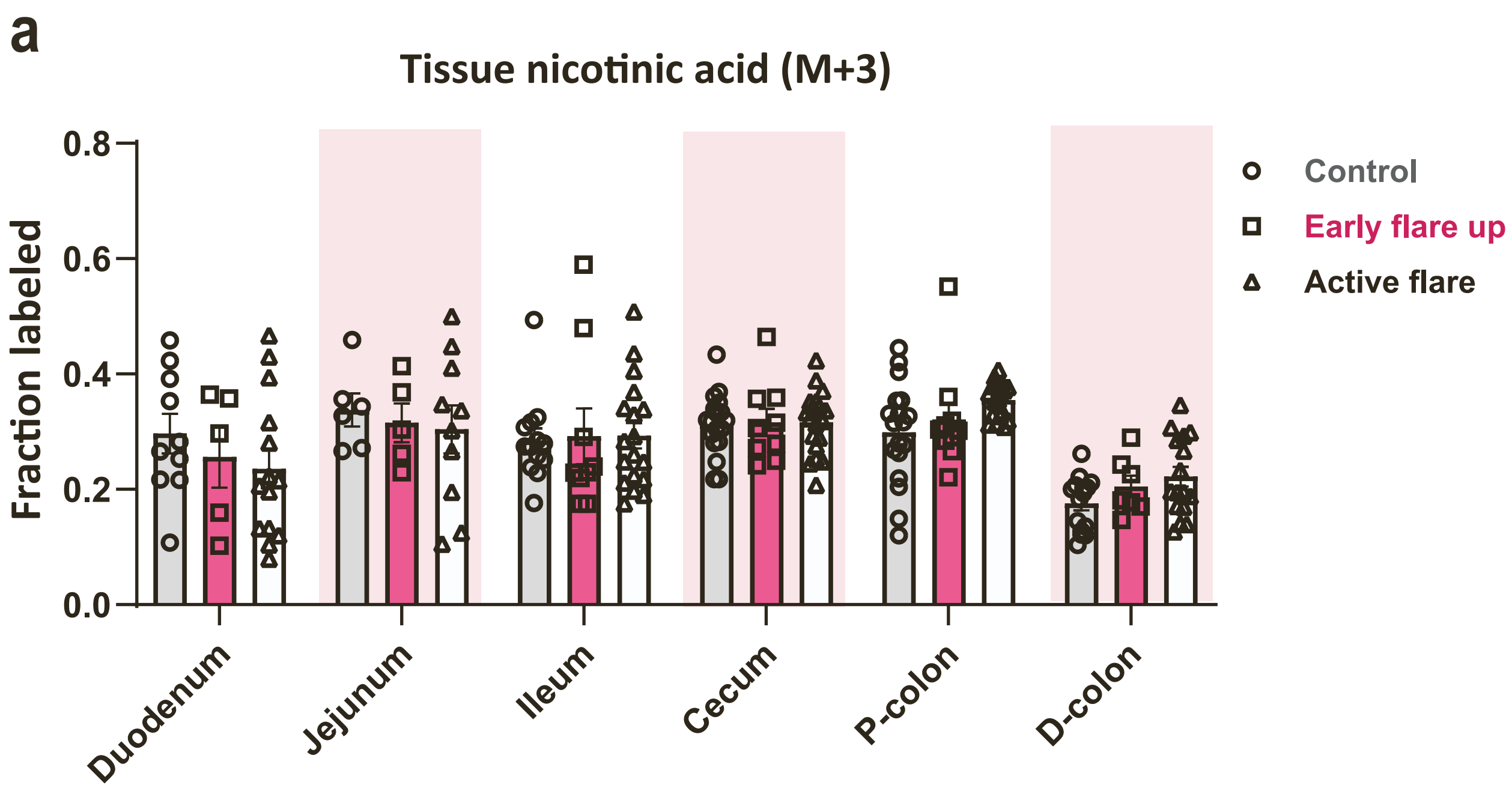
a
Tissue nicotinic acid (M+3)
Fraction labeled
0.0
0.2
0.4
0.6
0.8
Duodenum
Jejunum
Ileum
Cecum
P-colon
D-colon
Control
Early flare up
Active flare

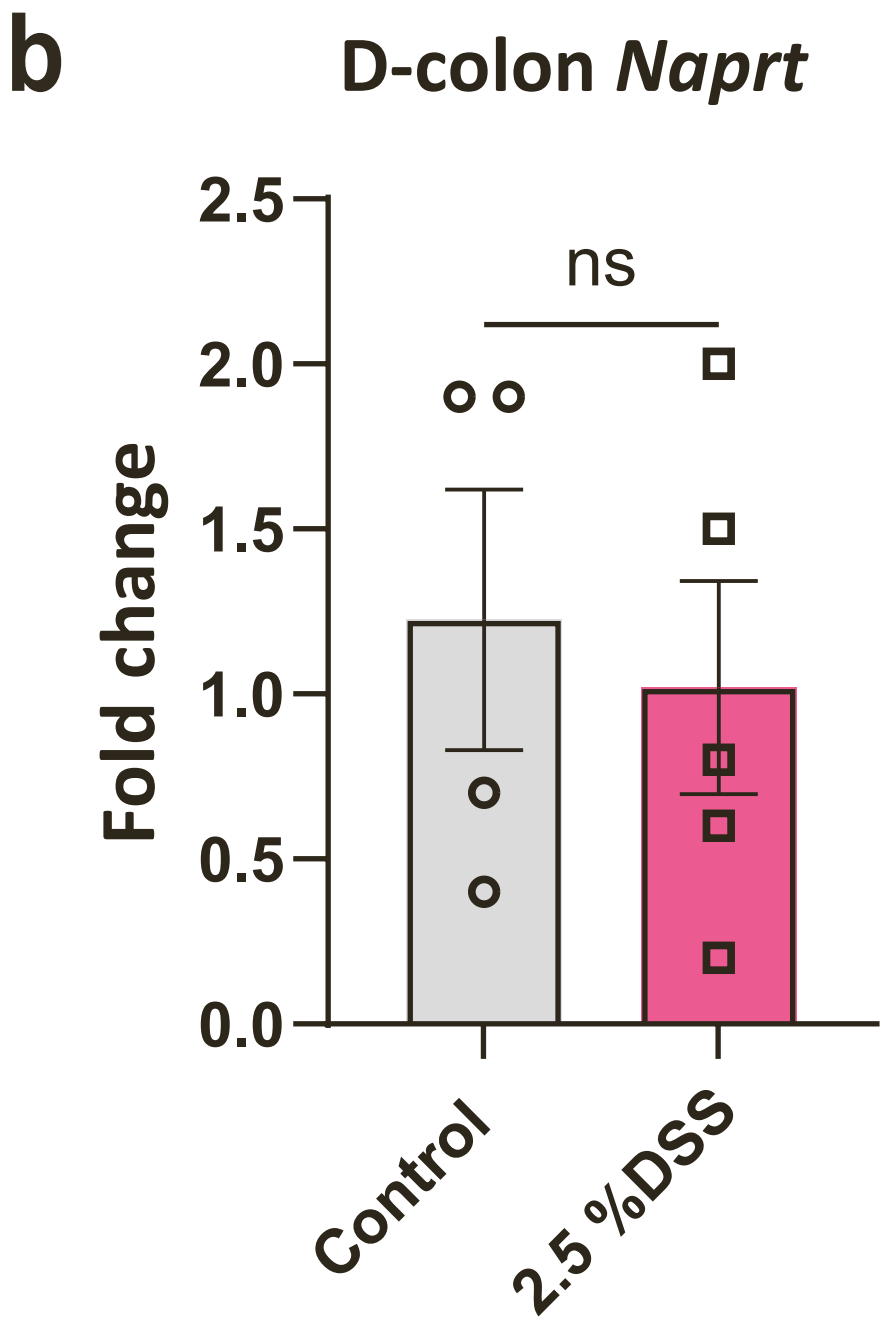
b
D-colon *Naprt*
Fold change
0.0
0.5
1.0
1.5
2.0
2.5
ns
Control
2.5 %DSS

Fig.S7| Flux from microbial nicotinic acid to host tissues.

(a) Tissues fractional labeled of nicotinic acid produced from microbial nicotinic acid (n=8-18). (**b**) qRT-PCR analysis of mRNA expression normalized to *Tbp* in the distal colon of nicotinate phosphoribosyltransferase (*Naprt*) during the active phase of DSS-induced colitis (days 8 and 11) compared to control (n=4-5). Data are presented as mean ± SEM. Statistical significance was determined by Mann-Whitney U test used for comparisons between two groups, and the Kruskal-Wallis test followed by Dunn's post hoc test for comparisons among more than two groups. ns= not significant.

Fig.S8

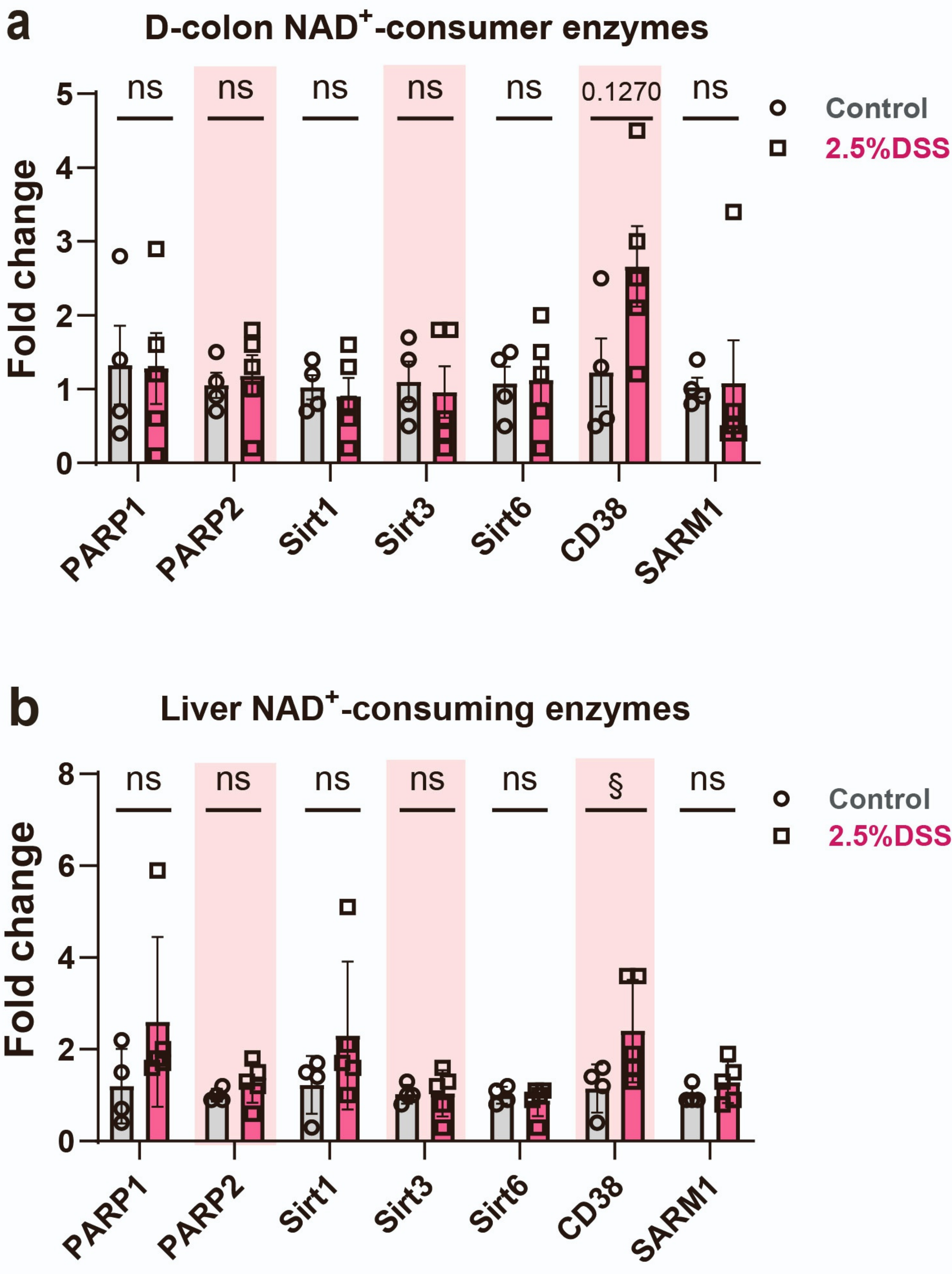
a
D-colon NAD+-consumer enzymes
Fold change
0
1
2
3
4
5
ns
ns
ns
ns
ns
0.1270
ns
Control
2.5%DSS
PARP1
PARP2
Sirt1
Sirt3
Sirt6
CD38
SARM1
b
Liver NAD+-consuming enzymes
Fold change
0
2
4
6
8
ns
ns
ns
ns
ns
§
ns
Control
2.5%DSS
PARP1
PARP2
Sirt1
Sirt3
Sirt6
CD38
SARM1

Fig.S8| Analysis of the activity of NAD^+-consuming enzymes during intestinal inflammation.

Analysis of mRNA expression measured by qRT-PCR and normalized to *Tbp* in tissues (**a**) distal colon and (**b**) liver; left to right, poly ADP-ribose polymerase (*PARP 1-2*), sirtuins (*Sirt 1,3,6*), *CD38,* sterile alpha and toll/interleukin-1 receptor motif-containing 1 (*SARM1*) during the active phase of DSS-induced colitis (days 8 and 11) compared to control. Data are presented as mean ± SEM, (n=4-5). Statistical significance was determined by Mann-Whitney U test used for comparisons between two groups. § <0.1, ns= not significant.

Supplemental table 1: Primers used for gene expression analysis

Target gene	Species	Taqman ID	RefSeq number
Ido1	Mouse	Mm00492590_m1	NM_001293690.1
Ido2	Mouse	Mm00524210_m1	NM_145949.2
Tdo2	Mouse	Mm01220281_m1	NM_019911.2
Nampt	Mouse	Mm00451938_m1	NM_021524.2
Nmnat1	Mouse	Mm01257929_m1	NM_133435.1
Nmnat2	Mouse	Mm00615393_m1	NM_175460.3
Nmnat3	Mouse	Mm00513791_m1	NM_144533.2
Nnmt	Mouse	Mm00447994_m1	NM_010924.2
Naprt	Mouse	Mm00553802_m1	NM_172607.3
Parp1	Mouse	Mm01321084_m1	NM_007415.2
Parp2	Mouse	Mm01319555_m1	NM_009632.2
Sirt1	Mouse	Mm01168521_m1	NM_001159589.1
Sirt3	Mouse	Mm00452131_m1	NM_001127351.1
Sirt6	Mouse	Mm01149042_m1	NM_001163430.1
Sarm1	Mouse	Mm00555617_m1	NM_001168521.1
CD38	Mouse	Mm01220904_m1	NM_007646.4
Il-6	Mouse	Mm00446190_m1	NM_031168.1
Il-1α	Mouse	Mm00439620_m1	NM_010554.4
Il-1β	Mouse	Mm00434228_m1	NM_008361.3
Tnf-α	Mouse	Mm00443258_m1	NM_001278601.1
Ifn-γ	Mouse	Mm01168134_m1	NM_008337.3
Il-10	Mouse	Mm00439614_m1	NM_010548.2
Il-22	Mouse	Mm01226722_g1	NM_016971.2
Lcn-2	Mouse	Mm01324470_m1	NM_008491.1
Ocln	Mouse	Mm00500910_m1	NM_008756.2
Tbp	Mouse	Mm00446971_m1	NM_013684.3

Supplementary table 2: Metabolite identification parameters.

Metabolite ID	Retention time (RT)	M/Z	Ionization mode
Tryptophan	6.658	205.0973	Positive ion mode [M+H]+
Kynurenine	6.74	209.0922	Positive ion mode [M+H]+
Kynurenic acid	4.847	190.0501	Positive ion mode [M+H]+
Nicotinamide	4.673	123.0553	Positive ion mode [M+H]+
Nicotinate	5.656	124.0394	Positive ion mode [M+H]+
NAD^+	8.193	664.1165	Positive ion mode [M+H]+
3-Hydroxyanthranilic acid	4.923	154.0499	Positive ion mode [M+H]+
Nicotinamide mononucleotide	7.691	335.064	Positive ion mode [M+H]+
Methyl nicotinamide	11.962	137.071	Positive ion mode [M+H]+
N-Me-4PY	4.551	153.0658	Positive ion mode [M+H]+
N-Me-6PY	4.794	153.0658	Positive ion mode [M+H]+
Quinolinate	10.762	166.014755	Negative ion mode [M-H]-
Quinaldic acid	5.354	174.055	Positive ion mode [M+H]+
Serotonin	6.849	177.1023	Positive ion mode [M+H]+
Tryptamine	5.608	161.1074	Positive ion mode [M+H]+
Xanthurenic acid	6.392	206.0452	Positive ion mode [M+H]+
5-Methoxytryptophan	6.869	235.1079	Positive ion mode [M+H]+
Anthranilate	6.898	138.055	Positive ion mode [M+H]+
Picolinic acid	7.843	124.0393	Positive ion mode [M+H]+
Indole	7.656	116.0507	Negative ion mode [M-H]-
Indole-3-propionic acid	4.992	188.0718	Negative ion mode [M-H]-
Indoleacetic acid	5.394	174.0561	Negative ion mode [M-H]-
Indolelactic acid	5.068	204.0668	Negative ion mode [M-H]-
Indole-3-acetylaldehyde	7.66	158.0613	Negative ion mode [M-H]-
